# Telmisartan induces browning of fully differentiated white adipocytes via M2 macrophage polarization

**DOI:** 10.1038/s41598-018-38399-1

**Published:** 2019-02-04

**Authors:** Eun Jeong Jeon, Dong Young Kim, Na Hyun Lee, Hye-Eun Choi, Hyae Gyeong Cheon

**Affiliations:** 1 Department of Pharmacology, Gachon University School of Medicine, Incheon, 21999 Republic of Korea; 2 Department of Health Sciences and Technology, GAIHST, Gachon University, Incheon, 21999 Republic of Korea

**Keywords:** Cell signalling, Obesity

## Abstract

Telmisartan is a well-known anti-hypertensive drug acting as an angiotensin 2 receptor blocker (ARB), but it also possesses partial PPARγ agonistic activity and induces insulin sensitivity. In the present study, we investigated the effects of telmisartan on macrophage polarization in association with its browning capacity, because PPARγ plays a key role in M2 polarization and in the browning of white adipocytes. Telmisartan induced M2 marker expression in murine macrophages concentration dependently, which was confirmed by flow cytometry. Both PPARγ and PPARδ activations appear to be responsible for telmisartan-induced M2 polarization. Telmisartan-treated conditioned medium (Tel-CM) of RAW264.7 cells and of bone marrow derived macrophages (BMDM) induced the expressions of browning markers in fully differentiated white adipocytes with reduced lipid droplets, and increased oxygen consumption rate and mitochondrial biogenesis. Levels of catecholamines (CA) released into the conditioned medium as well as intracellular tyrosine hydroxylase (TH) mRNAs were found to be increased by telmisartan, and browning effects of Tel-CM were lessened by β3 receptor antagonist (L-748,337), suggesting CA secreted into CM play a role in Tel-CM-induced adipocyte browning. Acute administration of telmisartan (2 weeks, *p.o*.) to C57BL/6J mice increased the expressions of browning markers and M2 markers in white adipose tissues, whereas macrophage depletion by clodronate liposome pretreatment attenuated the telmisartan-induced expressions of browning markers. Together, telmisartan was observed to induce the browning of fully differentiated white adipocytes, at least in part, via PPAR activation-mediated M2 polarization.

## Introduction

The renin-angiotensin system (RAS) plays key roles in the regulations of hydro-electrolyte balance and blood pressure^1^, and inhibition of this system using RAS blockers provides a therapeutic means of treating hypertension, which lead to the development of several RAS blockers^2^. The RAS is expressed in adipose tissues and influences adipocyte biology, and angiotensin 2 regulates adipocyte differentiation, adipokine secretion and glucose transport^3–5^. Two types of angiotensin 2 receptor exist, that is AT1 receptor and AT2 receptor, which differ in terms of expression and function^6^. Telmisartan (an RAS blocker) belongs to a family of AT1 receptor antagonists, and is widely used as an anti-hypertensive and anti-atherosclerotic drug. Unlike other AT1 receptor antagonists, telmisartan is also a partial PPARγ agonist^7^. PPARγ is a transcription factor that regulates the expressions of various target genes via heterodimer formation with retinoid X receptor (RXR), and thiazolidinediones are well known synthetic PPARγ agonists with insulin sensitizing effects^8^. As compared with full PPARγ agonists, partial PPARγ agonists, including telmisartan, ameliorate insulin resistance but have fewer side effects (e.g., less weight gain)^9^.

Macrophages are heterogeneous, and undergo mutual transition in response to various stimuli. Furthermore, changes in macrophage subpopulations play important roles in metabolic diseases^10,11^; for example, in obese animal models, macrophages (particular M1 proinflammatory phenotypes) infiltrate adipose tissues and induce low grade chronic inflammation and insulin resistance^12–14^. On the other hand, M2 polarized macrophages (or alternatively activated macrophages) appear to suppress obesity-induced inflammation^15^. Various signaling pathways (*i.e*. JNK, PI-3K/Akt, Notch, JAT/STAT signaling) are known to be implicated in M2 polarization^16^, and transcription factors, such as PPARγ and PPARδ, are known to play a key role in upregulation of M2 macrophage polarity^17,18^.

Recently, several reports provide evidence that “browning”, that is, the conversion of white to brown-like adipocytes might be a novel therapeutic strategy for the treatment of obesity^19^. It has also been reported M2 polarized macrophages induced the browning of differentiated white adipocytes via CA release independently of sympathetic nervous activation^20^. Telmisartan has been reported to promote energy expenditure, and concomitantly decreased adipocyte size and fat accumulation^21^, and to exert anti-inflammatory effects in macrophages and increase the expressions of M2 markers^22^. Accordingly, we investigated the potential involvement of M2 polarization in telmisartan-induced adipose tissue browning and mechanism responsible for the effects of telmisartan. We found telmisartan induced a shift in macrophage phenotypes toward M2 polarity and evoked the browning of white adipocytes. Moreover, PPARγ and PPARδ activations appeared to underlie telmisartan-induced M2 polarization.

## Results

### Telmisartan induced M2 polarization in RAW264.7 cells

**Figure 1:**
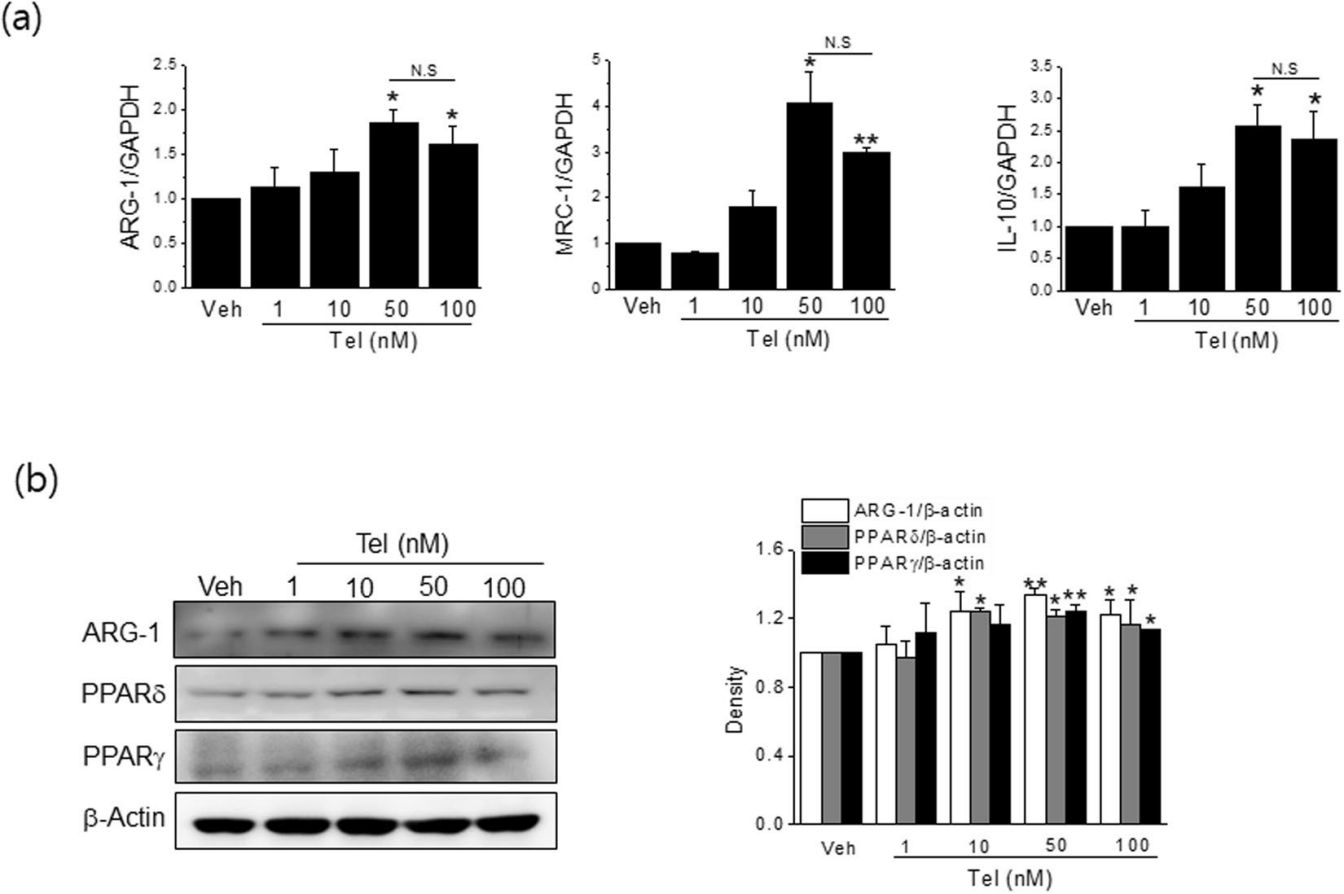
Telmisartan induced M2 polarization in RAW264.7 cells. RAW264.7 macrophages (3 × 10^5^ cells/well) were treated with various concentrations (1, 10, 50 and 100 nM) of telmisartan for 24 h, and then the expressions of M2 markers were determined by real time qPCR (**a**) and by western blot (**b**). Results are presented as means ± SDs of three experiments performed in duplicate. *p < 0.05, **p < 0.01 *vs*. vehicle. N.S; not significant.

**Figure 2:**
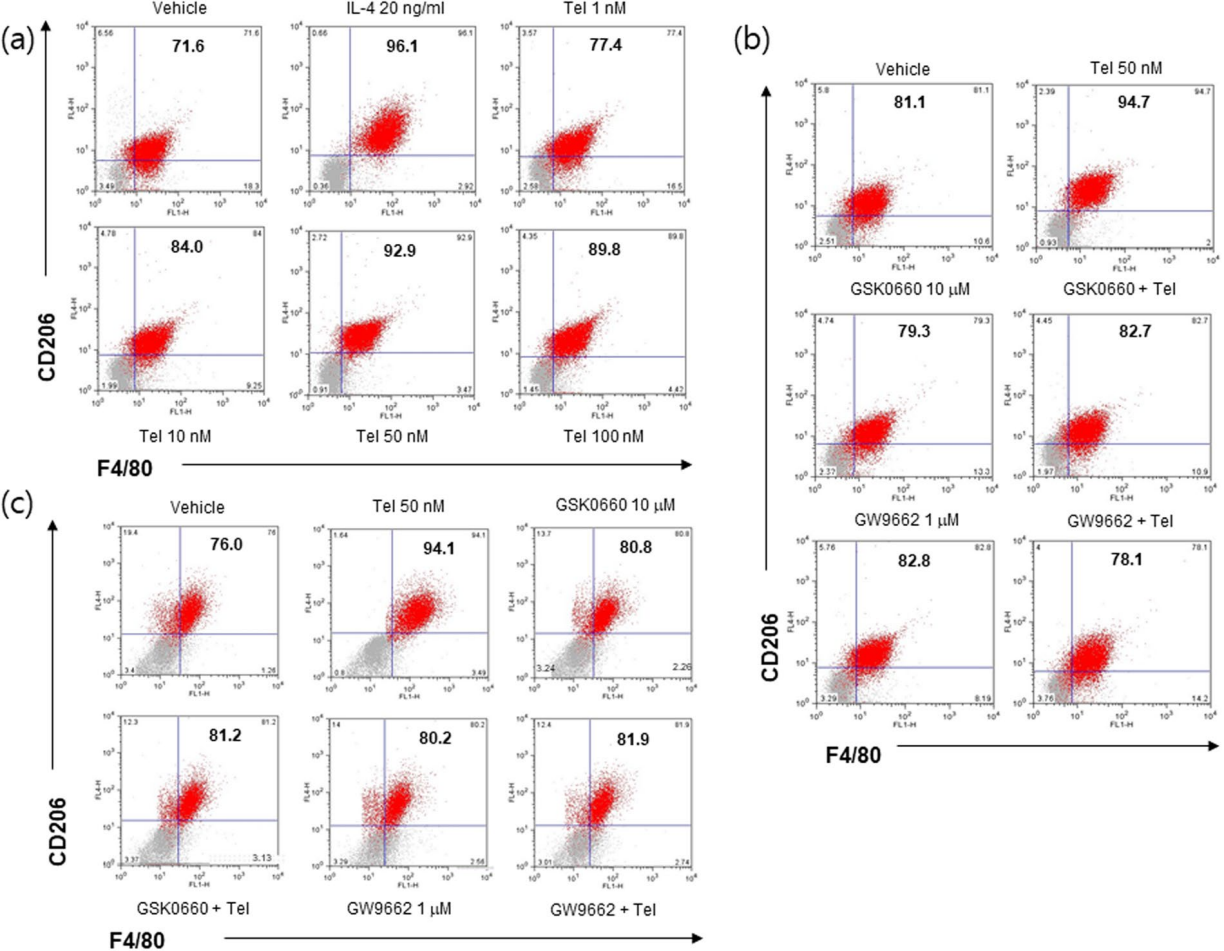
FACS analysis of telmisartan-induced M2 polarization. RAW264.7 macrophages (1 × 10^4^ cells/well) were treated with various concentrations (1, 10, 50 and 100 nM) of telmisartan for 24 h, and then flow cytometry was conducted using CD206 and F4/80 antibodies (**a**). RAW264.7 macrophages (**b**) or primary BMDM (**c**) were pretreated with PPARγ antagonist (GW9662, 1 μM) or PPARδ antagonist (GSK0660, 10 μM) for 1 h, and then treated with telmisartan (50 nM). Changes in the M2 population were determined by flow cytometry. IL-4 (20 ng/ml) was chosen as an M2 inducer. Representative results are shown.

We first examined the effects of telmisartan on M2 polarization in RAW264.7 cells (a murine macrophage cell line). Telmisartan concentration dependently induced the mRNA expressions of M2 markers (ARG-1, MRC-1 and IL-10) (Fig. 1a), and these expressions peaked at a concentration of 50 nM. Protein levels of ARG-1 (a representative M2 marker) were concomitantly increased by telmisartan (Fig. 1b). Consistently, flow cytometry with CD206 and F4/80 antibodies showed that telmisartan at 50 nM increased the percentage of RAW264.7 cells with the M2 phenotype (from 71.6% to 92.9% in vehicle and 50 nM telmisartan, respectively) (Fig. 2a). IL-4 (20 ng/ml) was used as a control M2 inducer, and it enhanced the M2 percentage to 96.1%.

### Mechanisms involved in telmisartan-induced M2 polarization

**Figure 3:**
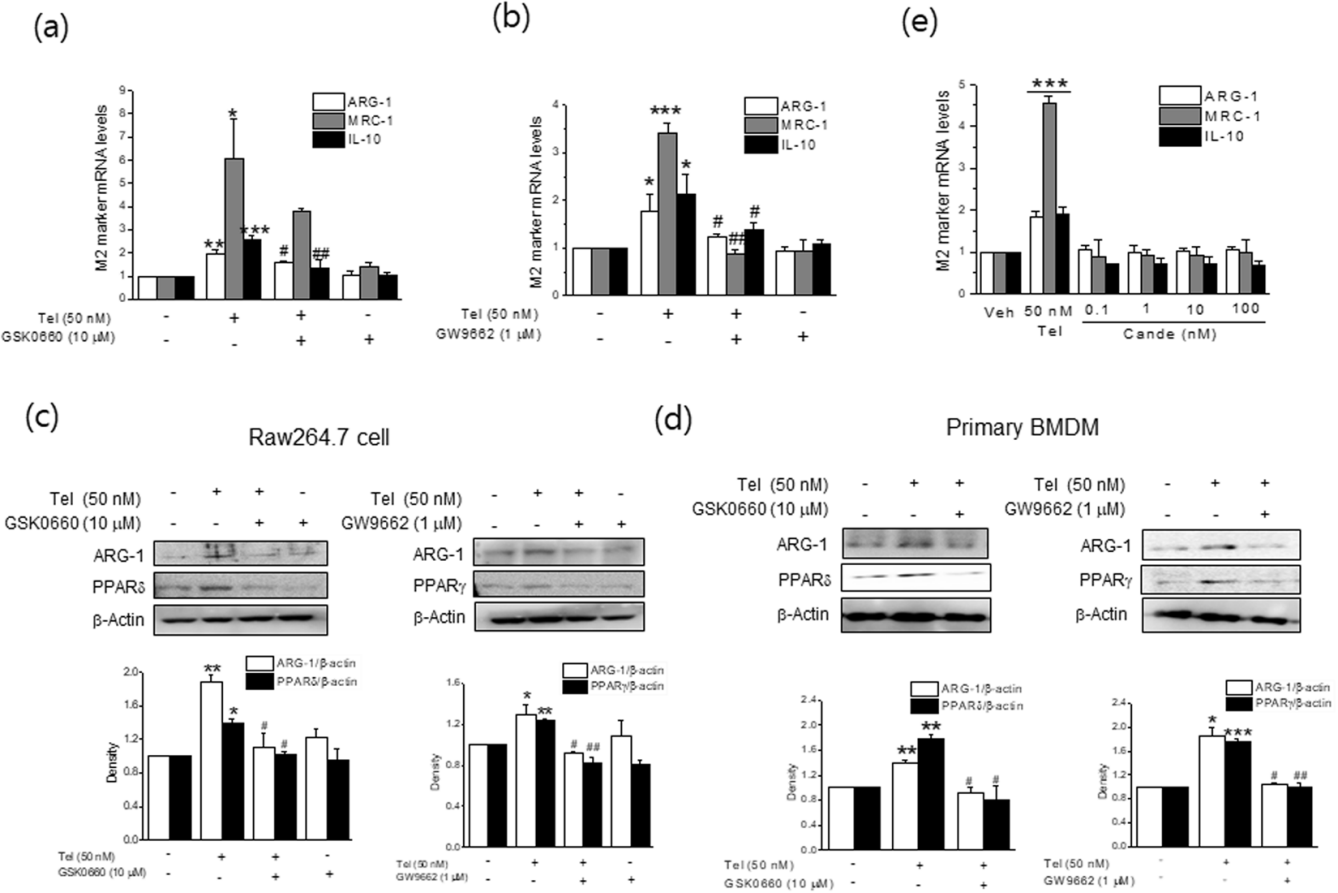
Effects of PPAR antagonists on telmisartan-induced M2 polarization. RAW264.7 macrophages (**a**–**c**) or primary BMDM (**d**) were pretreated with PPARγ antagonist (GW9662, 1 μM) or PPARδ antagonist (GSK0660, 10 μM) for 1 h, and then treated with telmisartan (50 nM) for 24 h. Expressions of M2 markers were determined by real time qPCR (**a**,**b**) and western blot (**c**,**d**). Separately, RAW264.7 cells were treated with candesartan (0.1–100 nM) for 24 h, and then expressions of M2 markers were determined by real time qPCR (**e**). Results are presented as the means ± SDs of three experiments performed in duplicate. *p < 0.05, **p < 0.01, ***p < 0.005 *vs*. vehicle. ^#^*p* < 0.05, ^##^*p* < 0.01 *vs*. telmisartan alone.

To investigate mechanisms underlying M2 polarization by telmisartan, we first examined whether PPAR activation was involved in telmisartain-induced M2 polarization. Telmisartan was found to increase PPARγ and PPARδ protein levels concentration dependently, as previously reported^7^ and its effect peaked at 50 nM (Fig. 1b). Next, RAW264.7 cells were pretreated with a PPARγ antagonist (GW9662, 1 μM) or a PPARδ antagonist (GSK0660, 10 μM) for 1 h, and the effects of telmisartan on M2 polarization were re-examined. Both antagonists suppressed the increased mRNA and protein expressions of M2 markers (Fig. 3a–c) and the proportions of M2 polarized cells by telmisartan were reduced in the presence of either antagonist (from 94.7% to 78.1% by GW9662; from 94.7% to 82.7% by GSK0660) (Fig. 2b), indicating that the activations of both PPARγ and PPARδ are required for telmisartan-induced M2 polarization. The involvement of PPAR activation in telmisartan-induced M2 polarization was further confirmed in primary BMDMs isolated from C57BL/6J mice (Figs 2c and 3d), by showing that both antagonists reduced M2 population as well as ARG-1 level as compared with telmisartan treatment alone.

On the other hand, candesartan, a well known ARB without PPAR agonistic activity was incapable of inducing expression of M2 markers, further supporting that telmisartan-induced M2 polarization was attributable to its activation of PPAR (Fig. 3e).

### Telmisartan-treated conditioned medium (Tel-CM) induced the expressions of browning markers in fully differentiated white adipocytes

**Figure 4:**
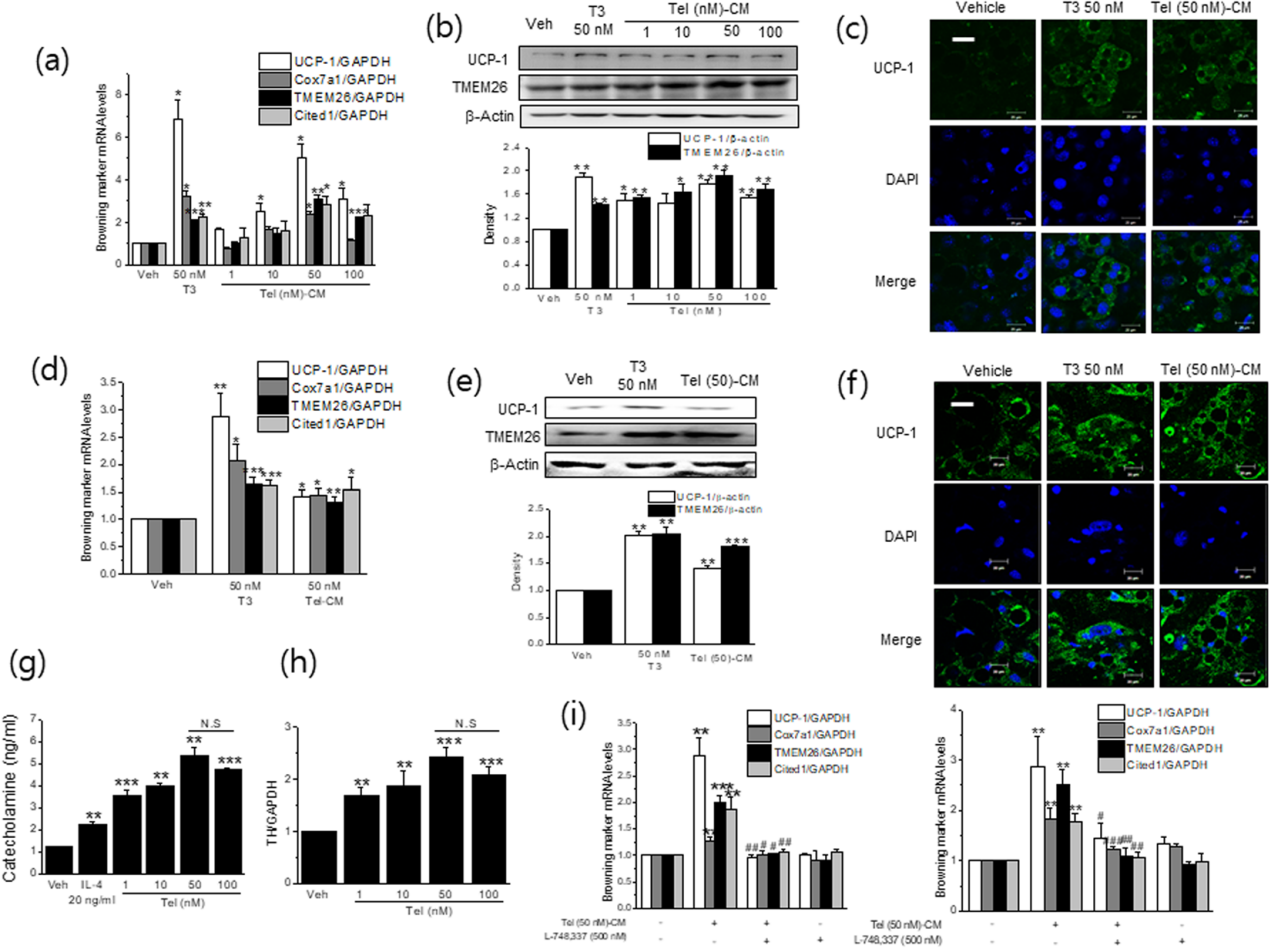
Tel-CM induced browning marker expression in differentiated white adipocytes. Differentiated 3T3/L1 adipocytes were treated with different concentrations of Tel treated-CM (400 μl) for 6 days (media were replaced with Tel-M2 CM every other day), and then the expressions of browning markers were determined by real time qPCR (**a**) and western blot (**b**). The immunostaining of UCP-1 is shown in (**c**) (*green labeling*, scale bar = 20 μm). Separately, primary adipocytes were treated with Tel-CM from primary BMDM for 6 days and then the expressions of browning markers were determined by real time qPCR (**d**) and western blot (**e**). The immunostaining of UCP-1 is shown in (**f**) (*green labeling*, scale bar = 20 μm). The levels of CA in conditioned medium (**g**) and intracellular TH mRNA (h) in RAW264.7 cells were measured by using assay kits and real time qPCR, respectively. L-748,337 (500 nM) was added into the culture medium every other day when white adipocytes were treated with Tel-CM, and then the expressions of browning markers were determined by real time qPCR (i left; 3T3/L1 adipocytes, i right; primary adipocytes). T3 (50 nM) was chosen as a positive control. Results are presented as the means ± SDs of three experiments performed in duplicate. *p < 0.05, **p < 0.01, ***p < 0.005 *vs*. vehicle. ^#^*p* < 0.05, ^##^*p* < 0.01 *vs*. Tel (50 nM)-CM alone. N.S; not significant.

Since M2 polarization was shown to elicit the browning of white adipocytes, we next evaluated the effects of Tel-CM (from RAW264.7 cells) on fully differentiated 3T3/L1 white adipocytes. Tel-CM induced the mRNA and protein levels of browning markers, including uncoupling protein-1 (UCP-1) (Fig. 4a,b), and these inductions peaked when cells were treated with 50 nM telmisartan-treated CM, which was in-line with maximum concentration for telmisartan-induced M2 polarization. Moreover, immunostaining for UCP-1 showed that Tel (50 nM)-CM increased UCP-1 expression as compared with vehicle-treated CM (Fig. 4c). Similar results were observed when differentiated primary adipocytes were exposed to primary macrophage CM treated with telmisartan (Fig. 4d–f). These results are consistent with previous studies, in which M2 CM was found to induce the browning of white adipocytes^20,23^. Triiodothyronine (T3, 50 nM) was used as a positive control for this experiment.

M2 macrophages are sources of CA that activate thermogenesis by binding to β3 adrenergic receptors in white adipocytes^20^. Thus, we assessed mRNA levels of intracellular TH, a rate determining enzyme for CA synthesis as well as levels of CA secreted into M2 CM after telmisartan treatment in RAW264.7 cells. We found telmisartan increased both TH expression and CA production as compared with vehicle (Fig. 4g,h). Furthermore, the addition of β3 receptor antagonist (L-748,337) reduced Tel-CM-induced browning (Fig. 4i left; 3T3/L1 adipocytes, right; primary adipocytes), which strongly suggested telmisartan-induced CA via M2 polarization may induce the browning of white adipocytes.

### Tel-CM induced browning in fully differentiated white adipocytes

**Figure 5:**
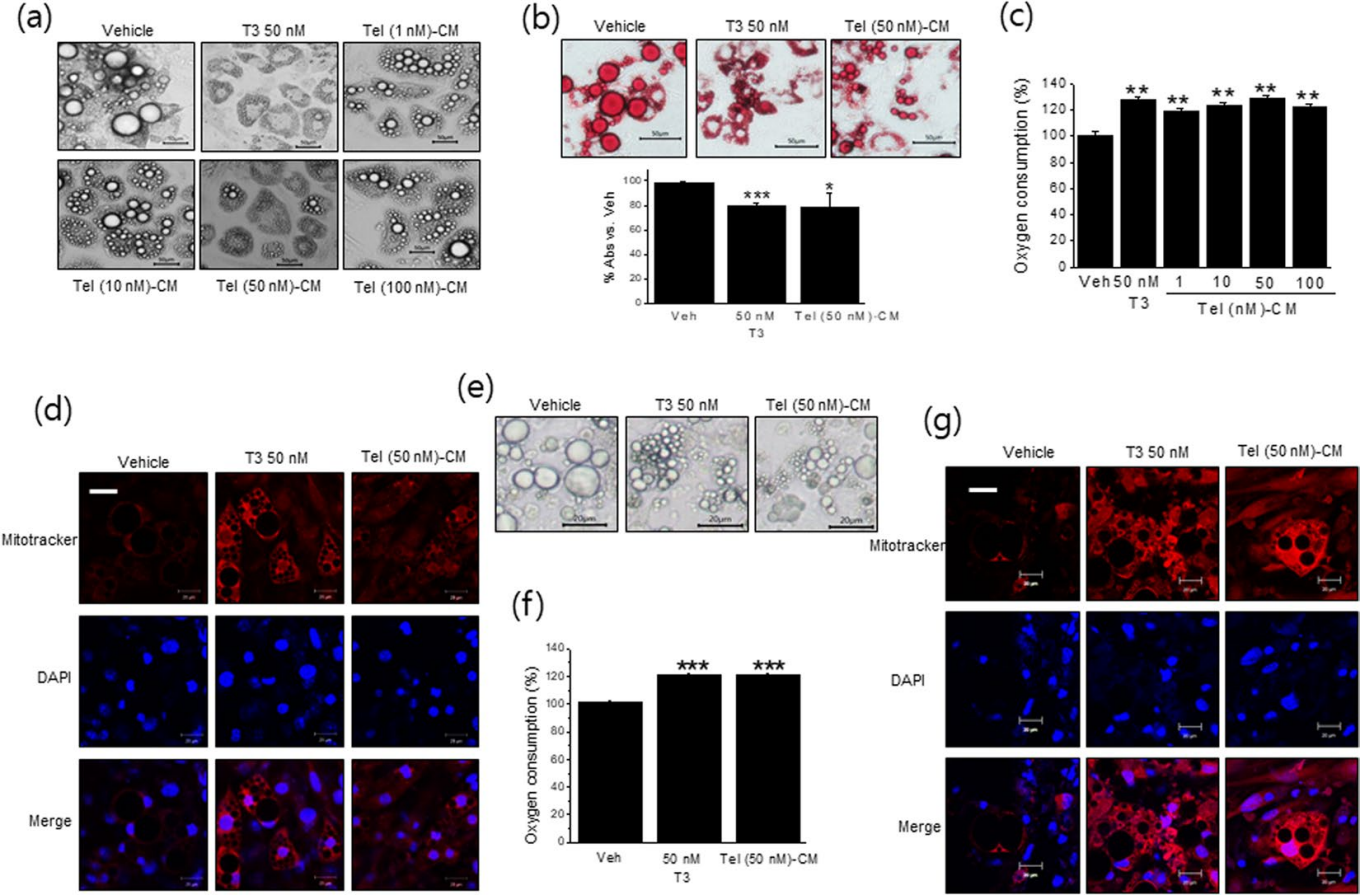
Effects of Tel-CM on the morphology of differentiated white adipocytes. Differentiated 3T3/L1 adipocytes were treated with different concentrations of Tel treated-CM (400 μl) for 6 days (medium changes were made every other day), and adipocyte morphology was then observed by microscopy (**a**, scale bar = 50 μm). The extent of lipid droplet accumulation was determined by Oil Red O staining (**b**, scale bar = 50 μm). OCR was determined using a Dissolved Oxygen meter and probe as described in Methods (**c**). Mitochondrial biogenesis was measured by Mitotracker staining (**d**) (*red labeling*, scale bar = 20 μm). Separately, primary adipocytes were treated with Tel-CM from primary BMDM for 6 days and then adipocyte morphology was observed by microscopy (**e**, scale bar = 20 μm). OCR was determined using a Dissolved Oxygen meter and probe (**f**). Mitochondrial biogenesis was measured by Mitotracker staining (**g**) (*red labeling*, scale bar = 20 μm). Results are presented as the means ± SDs of three experiments performed in duplicate. *p < 0.05, **p < 0.01, ***p < 0.005 *vs*. vehicle.

Consistent with the induction of browning markers by Tel-CM, treatment of 3T3/L1 adipocytes with Tel-CM for 6 days reduced lipid droplet sizes (Fig. 5a), and Tel-CM reduced lipid accumulation, also peaked at 50 nM, as determined by Oil Red O staining (Fig. 5b). These results further confirm that Tel-CM induced morphological change of white to brown-like adipocytes in parallel with the up-regulations of browning markers.

Oxygen consumption rate (OCR) was measured after treating 3T3/L1 adipocytes with Tel-CM for 6 days. As shown in Fig. 5c, OCR was increased by 130 ± 1.52% in Tel (50 nM)-CM treated cells, as compared with vehicle, which was in-line with the increased mitochondrial biogenesis observed by Mitotracker staining (Fig. 5d). All these results were confirmed when primary adipocytes were treated with Tel-CM obtained from primary BMDM (Fig. 5e–g).

### Telmisartan induced adipose tissue browning and M2 polarization *in vivo* at 23 °C

**Figure 6:**
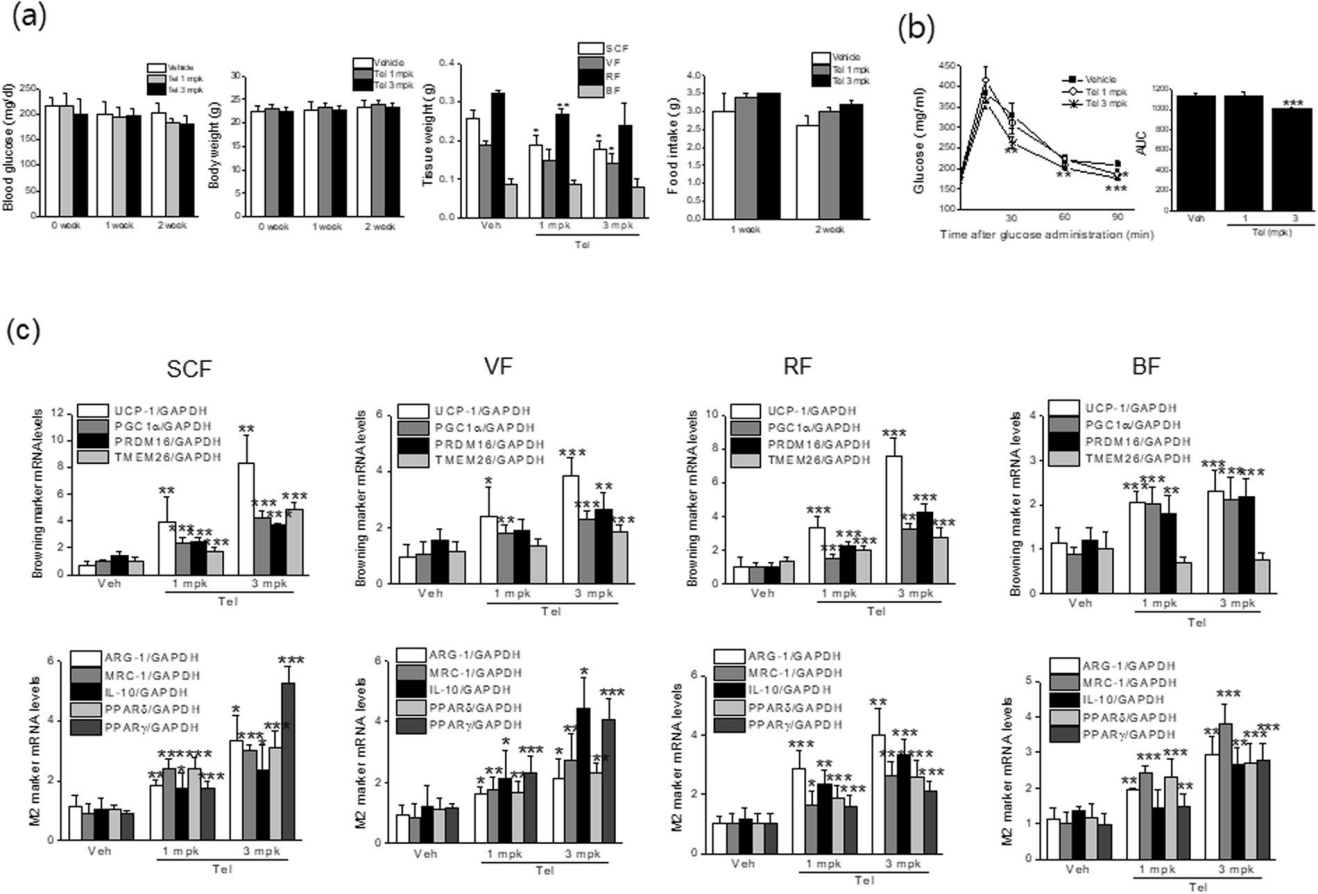
*In vivo* effects of telmisartan on body weights, glucose tolerance and mRNA marker expressions in adipose tissues at 23 °C. C57BL/6J mice (7 weeks old, male) were administered vehicle (0.9% saline) or telmisartan (1 or 3 mg/kg, *p.o*., once daily) for 2 weeks under constant conditions (temperature: 23 ± 2 °C, humidity: 40–60%, under a 12 h light/dark cycle). Body weights, fat weights, food intakes and nonfasting blood glucose levels were measured weekly (**a**). Oral glucose tolerance testing was performed after overnight fasting (**b**). The levels of browning markers and M2 markers in fats were determined by real time qPCR (**c**).

**Figure 7:**
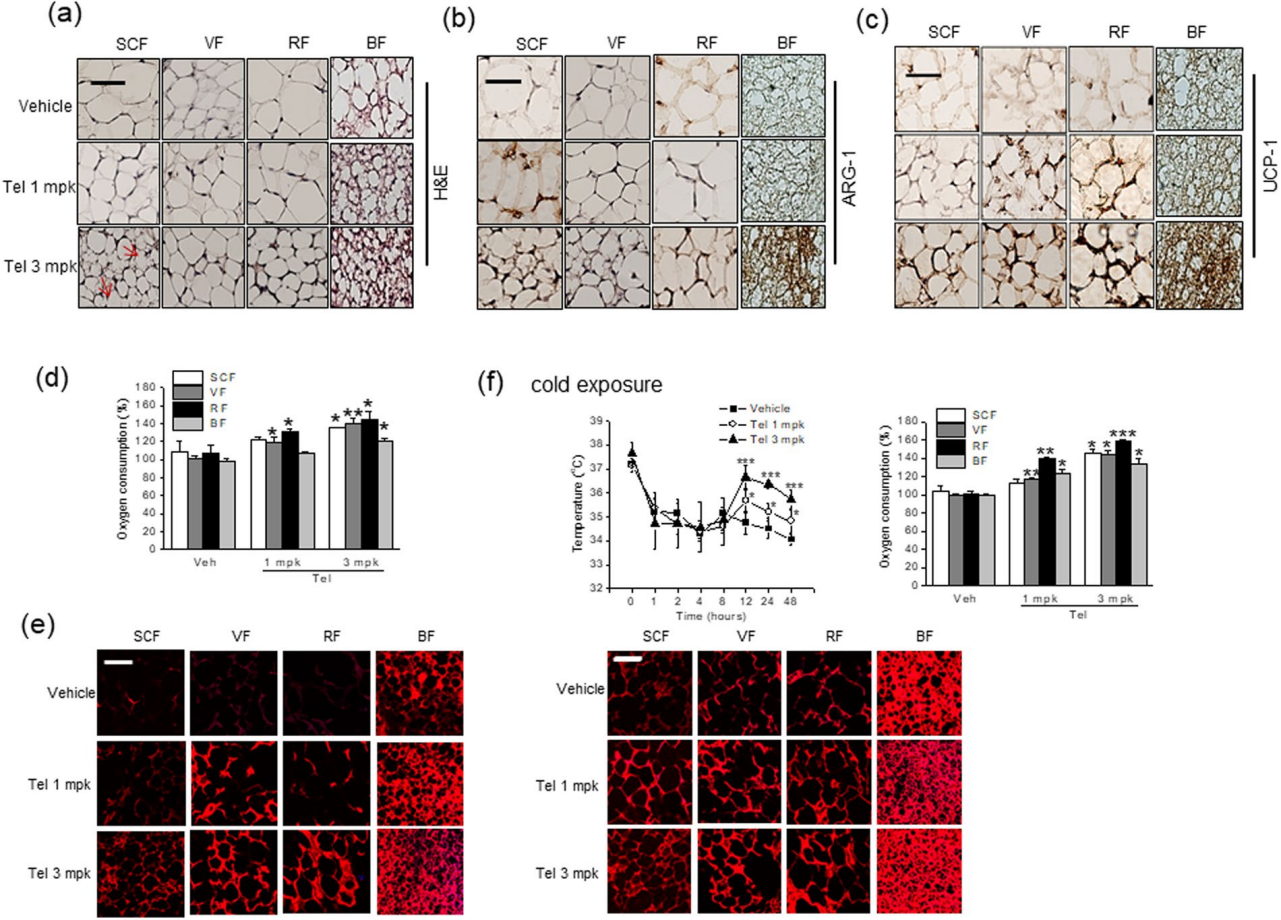
*In vivo* effects of telmisartan on adipose oxygen consumption rate and mitochondrial biogenesis at 23 °C. C57BL/6J mice (7 weeks old, male) were administered vehicle (0.9% saline) or telmisartan (1 or 3 mg/kg, *p.o*., once daily) for 2 weeks under constant conditions (temperature: 23 ± 2 °C, humidity: 40–60%, under a 12 h light/dark cycle). Adipose tissues were isolated and stained with hematoxylin and eosin (H&E) (**a**, scale bar = 20 μm), and ARG-1 and UCP-1 immunostained fat tissues were examined under a microscope (**b**,**c**, respectively) (scale bar = 20 μm, *brown labeling*). *Ex vivo* OCRs of various adipose depots were determined using a Mito-ID^®^ O_2_ extracellular sensor kit (**d**). Mitochondrial biogenesis was measured by Mitotracker staining (**e**) (*red labeling*, scale bar = 20 μm). Separately, telmisartan-treated mice were exposed to 4 °C for 48 h, and rectal temperature was measured at the indicated times (**f**). OCR was determined after exposure to 4 °C for 48 h, and mitochondrial biogenesis was measured by Mitotracker staining (**f**) (*red labeling*, scale bar = 20 μm). *p < 0.05, **p < 0.01, ***p < 0.005 *vs*. vehicle.

To confirm the M2 polarization-induced browning effects of telmisartan *in vivo*, we administered telmisartan (1 or 3 mg/kg, *p.o*., once daily) to C57BL/6J mice for 2 weeks under temperature of 23 ± 2 °C, and examined its effects on adipose tissue browning. Two weeks of treatment with telmisartan had little effect on nonfasting blood glucose levels or food intakes (Fig. 6a), which concurred with a previous report^5^, but at 3 mg/kg of telmisartan improved glucose clearance, as determined by oral glucose tolerance test (OGTT) (Fig. 6b). Although little change in body weights was observed, white adipose tissue masses were reduced upon telmisartan administration (Fig. 6a). As shown in Fig. 6c, telmisartan induced browning marker expressions including UCP-1, and concomitantly increased mRNA levels of M2 markers (ARG-1, MRC-1, and IL-10) and of PPARs in subcutaneous fats (SCF). Thus, telmisartan appeared to promote M2 polarization, in parallel with increasing browning marker expression *in vivo*. Remarkably, increased browning marker expression as well as M2 markers and PPARs were also observed in visceral (VF) and reproductive fats (RF) (Fig. 6c), which are relatively resistant to browning. Correspondingly, adipose tissues obtained from telmisartan-treated animals showed reduced adipocyte size with increased multilocular and UCP-1 positive cells (Fig. 7a,c, indicated as arrows) and increased ARG-1 immunostaining (Fig. 7b). Congruent with increased OCR *ex vivo* by ~135–144% in white adipose tissues and increased mitochondrial biogenesis (Fig. 7d,e), telmisartan-treated mice were resistant to the reduction of body temperature upon exposure to 4 °C for 48 h (Fig. 7f), in which OCR *ex vivo* was further enhanced by ~143–168% in white adipose tissues with increased Mitotracker staining (Fig. 7f). Together, these results suggest telmisartan induces adipose tissue browning *in vivo* via M2 polarization, resulting in the increases of energy expenditure. Separately, increased expression of thermogenic genes and M2 markers as well as increased *ex vivo* OCR by ~120% were observed in brown adipose tissues of telmisartan-treated group (Fig. 7), and this increase of OCR and mitochondrial biogenesis was more noticeable after cold exposure (Fig. 7f), suggesting that telmisartan may also activate brown adipocytes in a direct manner.

### Telmisartan induced adipose tissue browning and M2 polarization *in vivo* at thermoneutral condition

**Figure 8:**
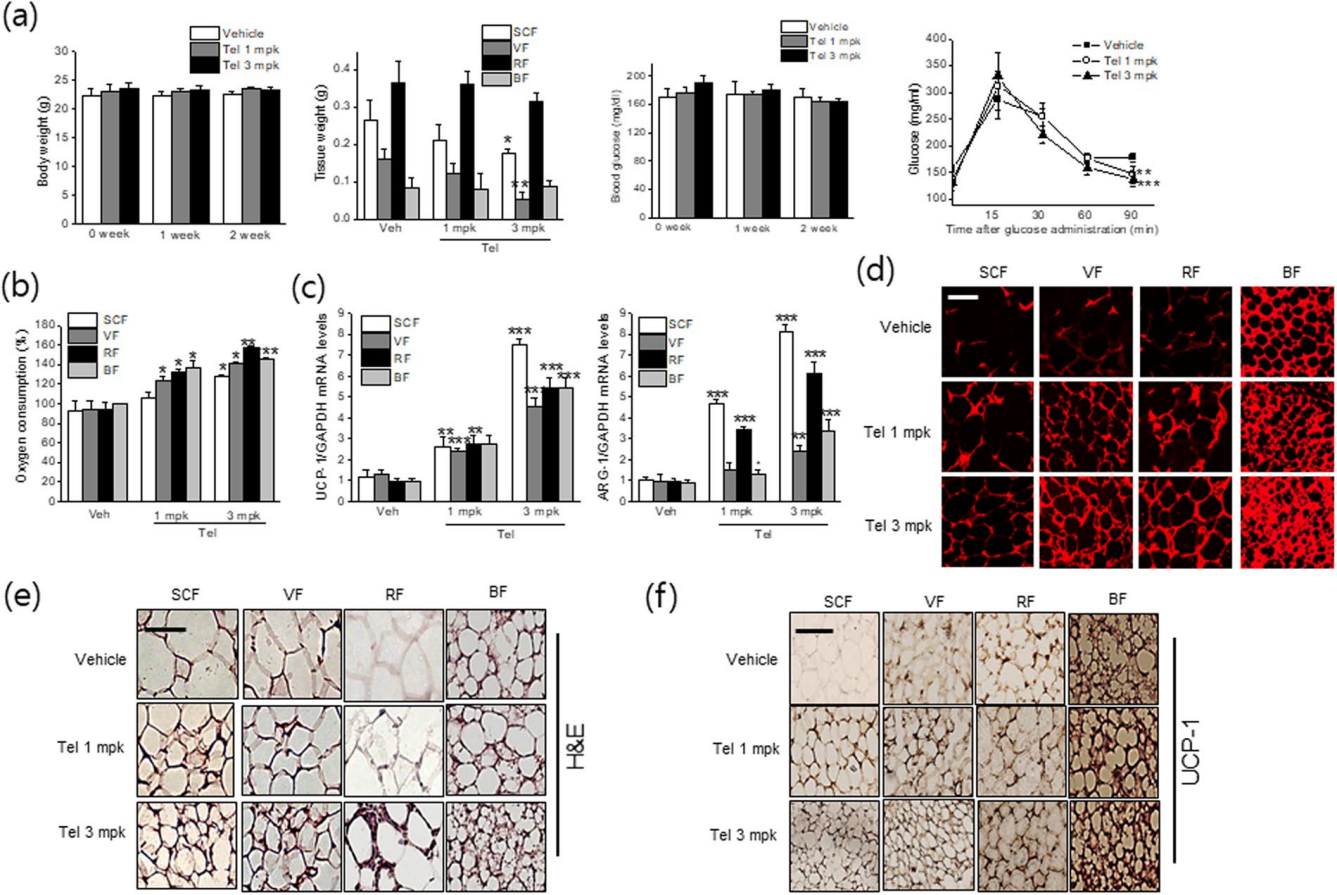
*In vivo* browning and M2 polarization effects of telmisartan at 30°C. C57BL/6J mice (7 weeks old, male) were administered vehicle (0.9% saline) or telmisartan (1 or 3 mg/kg, *p.o*., once daily) for 2 weeks under constant conditions (temperature: 30 ± 2 °C, humidity: 40–60%, under a 12 h light/dark cycle). Body weights, adipose tissue weights and nonfasting blood glucose levels were measured weekly. Oral glucose tolerance testing was performed after overnight fasting (**a**). *Ex vivo* OCRs of various adipose depots were determined using a Mito-ID^®^ O_2_ extracellular sensor kit (**b**). The levels of UCP-1 and ARG-1 in fats were determined by real time qPCR (**c**). Mitochondrial biogenesis was measured by Mitotracker staining (**d**) (*red labeling*, scale bar = 20 μm). Adipose tissues were stained with hematoxylin and eosin (H&E) (**e**), and UCP-1 immunostained fat tissues were examined under a microscope (**f**) (scale bar = 20 μm, *brown labeling*). *p < 0.05, **p < 0.01, ***p < 0.005 *vs*. vehicle.

Since housing temperature of 23 °C is known to impose thermal stress, the browning effects of telmisartan were re-evaluated at thermoneutral condition, in which telmisartan was given to thermoneutral mice, being maintained at temperature of 30 ± 2 °C during *in vivo* study. In a manner similar to the results obtained under the temperature of 23 °C, browning effects of telmisartan were also evident even at thermoneutral condition, as white adipose tissue masses were lower (Fig. 8a) with reduced adipocyte sizes (Fig. 8e), concurrent with improved glucose tolerance (Fig. 8a), increased OCR (Fig. 8b), increased UCP-1 and ARG-1 expression (Fig. 8c,f), and increased mitochondrial biogenesis (Fig. 8d).

### Effects of macrophage depletion in the browning action of telmisartan

**Figure 9:**
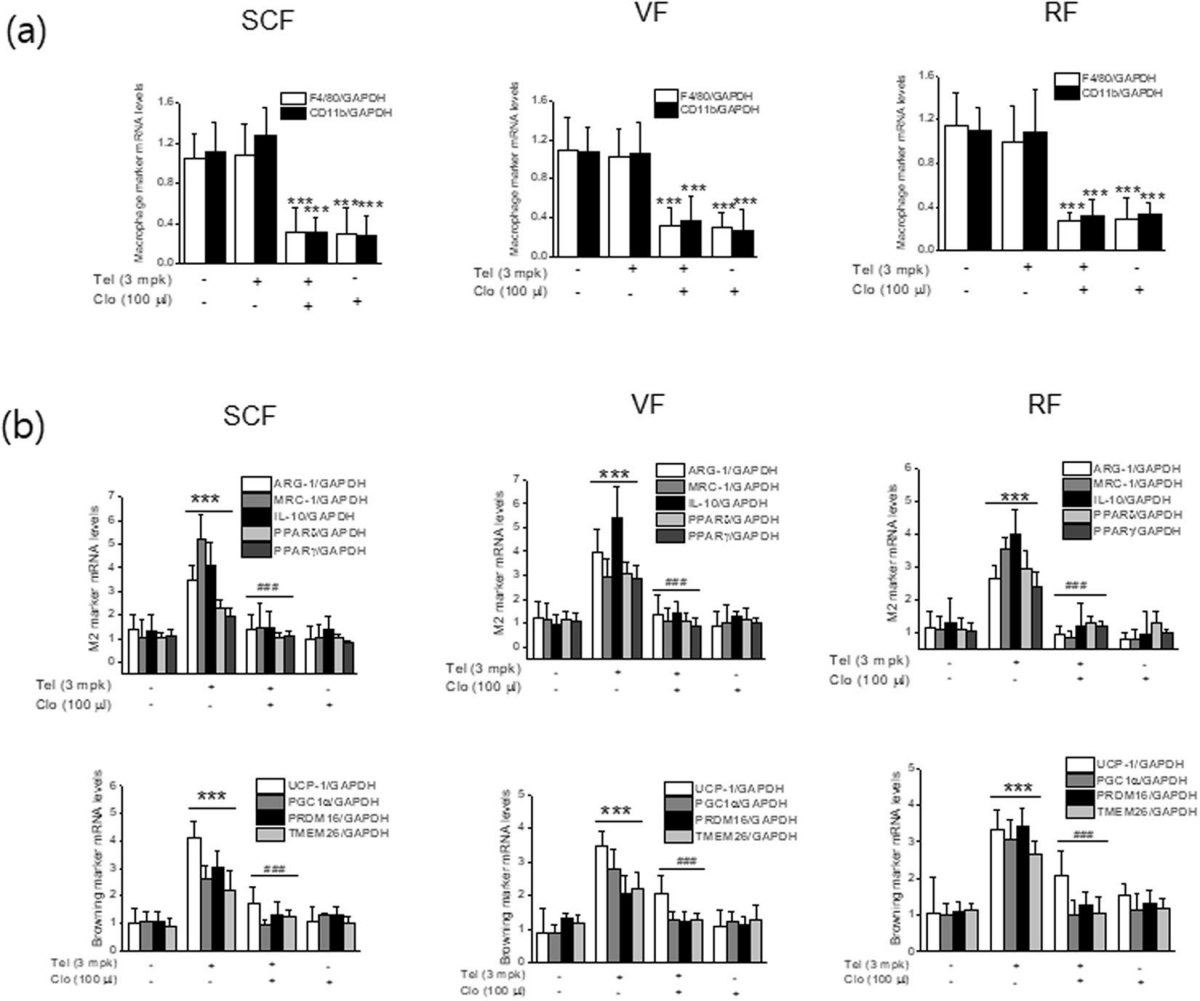
Effects of macrophage depletion in the browning action of telmisartan. C57BL/6J mice (7 weeks old, male) were treated with clodronate liposomes (every 2 days, *i.p*., 100 μl, n = 8) or control liposomes (n = 8) every other day and with telmisartan (3 mg/kg, *p.o*., once daily for 2 weeks). Extents of macrophage depletion in adipose tissues were assessed by real time qPCR for the expressions of F4/80 and CD11b (**a**), and the expressions of browning markers, M2 markers and PPARs were determined by real time qPCR (**b**).

**Figure 10:**
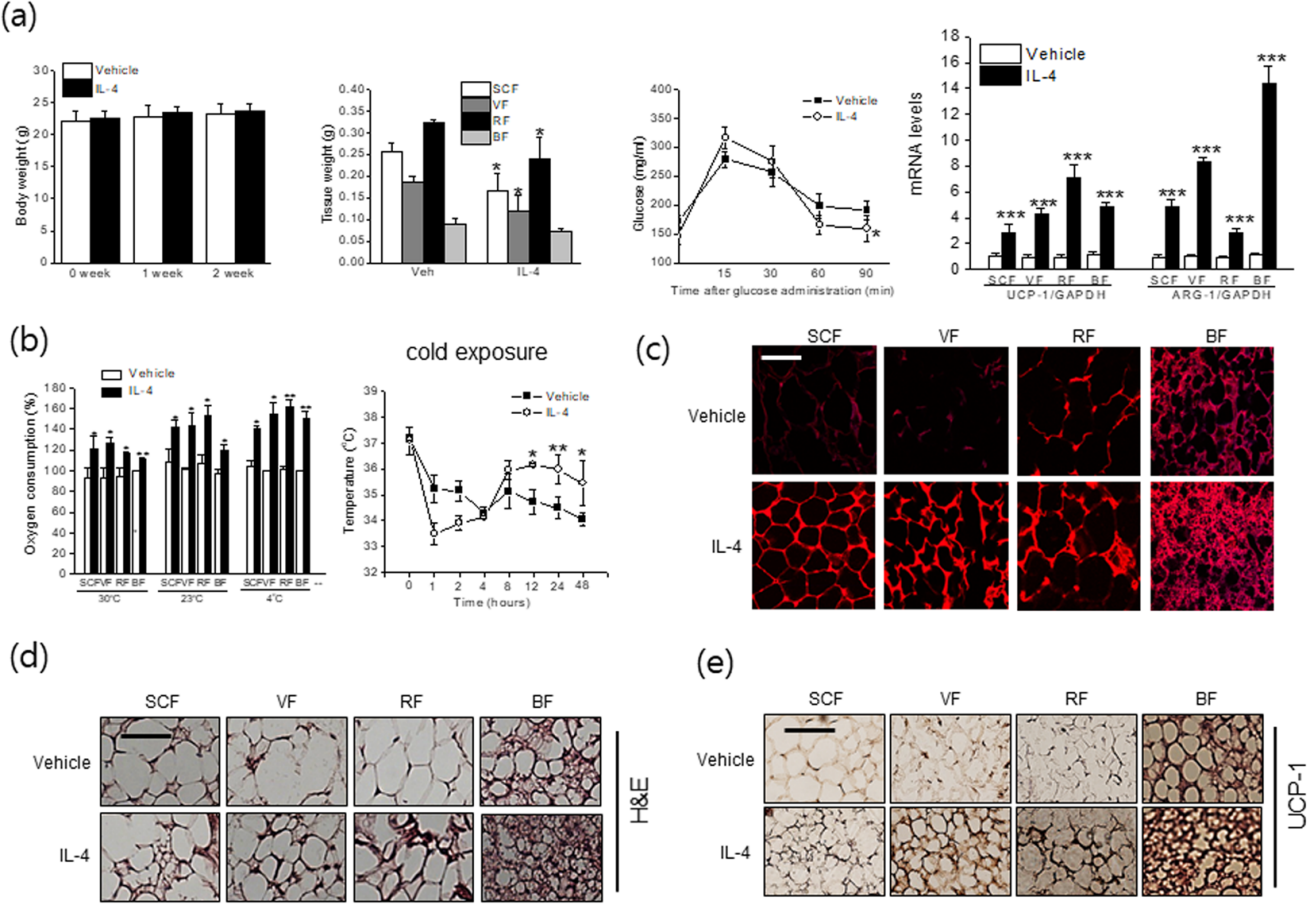
*In vivo* browning and M2 polarization effects of IL-4. IL-4 complexed with anti-IL-4 antibody (1:5) was intraperitoneally administered every other day for 2 weeks under temperature of either 23 ± 2 °C or 30 ± 2 °C, and body weights, fat weights, and blood glucose levels were measured. The mRNA levels of UCP-1 and ARG-1 were determined by real time qPCR (**a**). E*x vivo* OCRs of various adipose depots were determined using a Mito-ID^®^ O_2_ extracellular sensor kit, using tissues obtained from telmisartan-treated mice at 30 °C and 23 °C (**b**). Upon cold exposure for 48 h, rectal temperatures of mice treated at 23 °C were measured, after which isolated tissues were subjected to OCR measurement (**b**). Representative results of Mitotracker staining, H&E staining and UCP-1 immunostaining of adipose tissues treated with IL-4 complex under temperature of 23 ± 2 °C were shown (**c–e**). *p < 0.05, ***p < 0.005 *vs*. telmisartan with control liposome, ^###^*p* < 0.005 *vs*.vehicle.

To confirm M2 polarization is critical for telmisartan-induced browning, C57BL/6J mice were administered clodronate liposomes (100 μl, every other day for 2 weeks) before telmisartan, and the expressions of browning markers in adipose tissues were then re-examined. The extent of macrophage depletion by clodronate was confirmed by real time qPCR for F4/80 and CD11b in adipose tissues (Fig. 9a). Cotreatment with clodronate liposome and telmisartan was found to reduce the expressions of M2 markers and PPAR, concomitant with decreased browning markers as compared with telmisartan alone in various adipose depots (Fig. 9b). To further address the roles of M2 polarization on adipose tissue browning, type 2 cytokine IL-4, a well-known M2 inducer was examined for its browning effects under the same experimental condition (temperature of 23 ± 2 °C), which showed that IL-4 indeed induced expression of ARG-1 as well as UCP-1 (Fig. 10a,e), in association with increased OCR (Fig. 10b) and mitochondrial biogenesis (Fig. 10c), and reduced adipocyte sizes and adipose tissue weights (Fig. 10a,d). As shown in Fig. 10b, the effects of IL-4 on OCR were more evident at colder temperature than themoneutral condition. Furthermore, treatment with IL-4 into thermoneutral mice also increased UCP-1 expression (results not shown), in agreement with previous results^24^. These results confirm telmisartan induced the browning of white adipocytes at least in part via M2 polarization possibly induced by the activation of both PPARγ and PPARδ.

## Discussion

**Figure 11:**
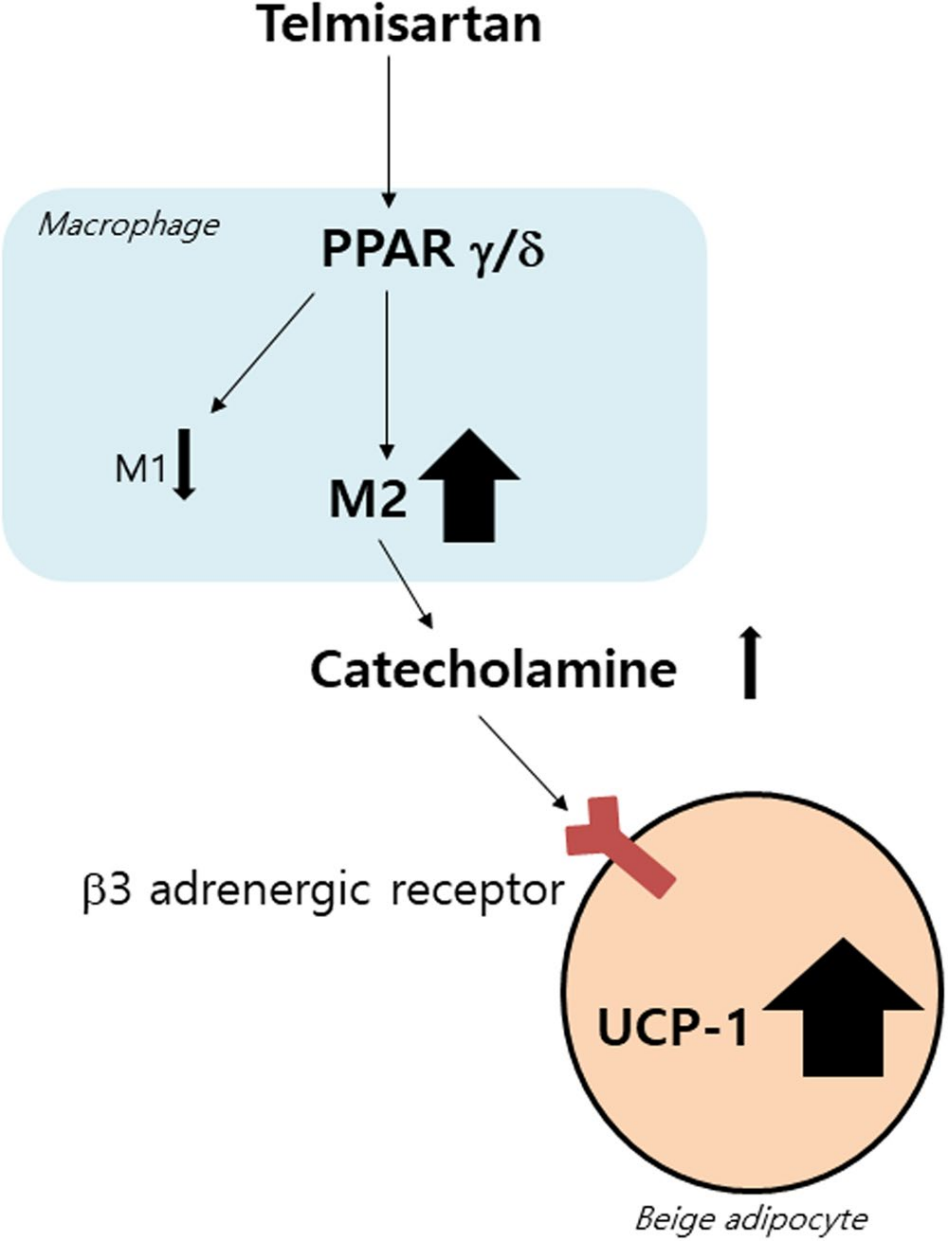
A proposed working model of telmisartan’s browning action.

In the present study, telmisartan was found to elicit M2 macrophage polarization via PPARγ and PPARδ activation in murine macrophages, and M2 conditioned medium obtained from telmisartan treated macrophages (Tel-CM) was observed to induce the browning of differentiated white adipocytes. The observations made after telmisartan administration to C57BL/6J mice supported these *in vitro* findings, as the expressions of browning markers and M2 markers were elevated in the adipose tissues of telmisartan administered animals. More importantly, *in vivo* macrophage depletion by clodronate attenuated the telmisartan-induced upregulation of browning marker expression in adipose tissues. In aggregate, our results provided novel evidence that the browning inducing effects of telmisartan are, at least in part, attributed to macrophage polarization to the M2 phenotype via PPARγ and PPARδ activation. A proposed working model of telmisartan’s browning action is presented in Fig. 11.

Dysregulation of the RAS contributes to the pathogenesis of cardiovascular diseases including atherosclerosis and hypertension. Recent studies have demonstrated that RAS also acts to control energy homeostasis^5,21^. Angiotensin 2, a product of RAS, is produced by white adipocytes^25^, and its production has been reported to be positively correlated with obesity^26^. Angiotensin 2 acts through AT1 and AT2 receptors, and *in vitro* and *in vivo* studies have revealed AT1 receptor KO mice exhibit thermogenic gene upregulation, in association with appearance of multilocular lipid droplets, indicating angiotensin 2 regulates energy expenditure mainly via AT1 receptor^27^.

Furthermore, pharmacological regulation of RAS has demonstrated therapeutic potential for the treatment of obesity and its related metabolic disorders. For example, losartan, a well-known AT1 receptor antagonist, increased energy expenditure by inducing adipocyte browning, and reduced body weights and increased insulin sensitivity in diet-induced obese mice^28^. Likewise, telmisartan was also reported to attenuate diet-induced weight gain and to promote the differentiation of small adipocytes and energy expenditure^21,29^. Many possible pathways related to telmisartan action appear to be PPARδ dependent^30^ but AT1 receptor independent^31^. In addition, other studies have reported telmisartan increased UCP-1 expressions in both white and brown adipose tissues and that these up-regulations were accompanied by increased OCR and energy expenditure^5,32^, which may be mediated by maintaining phosphorylation of PPARγ at ser112. In our experimental conditions, telmisartan also directly induced browning when administered to differentiated 3T3/L1 adipocytes (results not shown).

Recently, browning, that is, white to brown-like adipocyte conversion, is considered an attractive therapeutic strategy for the treatment of human obesity^19^. Brown-like adipocyte can be induced under different conditions, and this conversion is associated with decreased body weights and insulin resistance via non-shivering thermogenesis^33,34^. Accordingly, the pharmacological manipulation of browning may provide a means of influencing overall energy metabolism^35^. The most prominent characteristics of browning are the induction of UCP-1 expression and multiple mitochondria, and the presence of multilocular lipid droplets. Up to now, various transcription regulators, secreted mediators and proteins have been shown to be involved in the browning process, and representative examples are PRDM16, PPARγ and PGC1α and so on. In addition to many secretory molecules such as type 2 cytokines (IL-4 and IL-13), adiponectin, irisin, FGF21, cardiac peptide and prostaglandin, CA which are released from activated sympathetic nerve terminals are well-known stimulators of browning^24,34,36,37^. In the present study, CA in Tel-CM appeared to be important for the browning action of telmisartan, as telmisartan increased intracellular TH mRNA expression and CA production from RAW264.7 cells in association with the macrophage polarity switch to the M2 phenotype. Moreover, the presence of L-748,337, a β3 receptor antagonist lessened the Tel-CM-induced browning effect, implying that CA in Tel-CM play a key role in the browning.

Macrophages populations are heterogenous, have multiple functions and are essential component of innate immunity^10,11^, and macrophage infiltration into adipose tissue is a common feature of obesity. In particular, proinflammatory M1 macrophages play a role in obesity-driven low grade chronic inflammation, and consequent insulin resistance^12–14^. Recently, the importance of M2 polarization in adipose tissue browning was made evident by several studies, in which CA released from M2 macrophage were found to stimulate thermogenesis via β3 adrenergic receptor activation^20^. Fujisaka *et al*. reported telmisartan altered M1 and M2 markers in high fat diet-induced mice, and decreased the M1 to M2 ratio^22^. In the present study, telmisartan increased M2 macrophage polarization, and Tel-CM effectively induced browning markers in fully differentiated 3T3/L1 white adipocytes. Consistent with previous reports^22^, the mRNA expression of LPS-induced M1 markers was also reduced by telmisartan (results not shown). Interestingly, Liu *et al*. noticed M2 macrophage conditioned medium driven by RIP140 knockdown also triggered white adipocyte browning^23^. These results indicate telmisartan is capable of inducing white adipocyte browning, at least in part, via M2 polarization. However, its relative contribution to telmisartan-induced adipocyte browning as compared with direct browning effects is unclear. A recent report contradicted the proposed role of M2 macrophages in adipose tissue thermogenesis^38^, and this discrepancy can only be clarified by additional study.

Several signaling pathways direct macrophage plasticity and polarization, and several transcription factors also play key roles. PPARδ and PPARγ, which are ligand-activated transcription factors, control macrophage polarization when induced by IL-4 or IL-13. In fact, PPARδ or PPARγ deficient macrophages have been shown to resist M2 polarization^17,39^, and possible crosstalk between PPAR and the IL-4-STAT6 pathway may be involved in the M2 polarization. Since other ARBs without PPARγ agonistic activity (*e.g*., candesartan) do not cause M2 polarization^22^, which was confirmed in our experimental condition, the macrophage polarizing effects of telmisartan appear to be due to PPARγ activation, and not to angiotensin blockade *per se*. With regard to the protection afforded by telmisartan against acute myocardial infarction, high levels of M2 macrophages were observed within the cardiac specimens of Zucker Diabetic Fatty Rats^40^. In another study, telmisartan was observed to attenuate brain inflammation, via M2 microglia polarization, mainly by CAMMKβ dependent AMPK activation, and partly by PPARγ activation^41^. These results are consistent with our current findings that telmisartan via the activation of PPARγ and PPARδ is capable of inducing M2 polarization, and that this contributes to the browning of white adipocytes. On the other hand, neither PPARγ nor PPARδ antagonist completely reversed telmisartan-induced M2 polarization, which suggests mechanisms other than PPAR activation also contribute to the effects of telmisartan. Given previous observations that telmisartan activated AMPK in skeletal muscle and adipose tissue^42,43^, it may be that alternative means of AMPK activation are involved in telmisartan-induced M2 polarization in macrophages.

The *in vivo* anti-obesity effects of telmisartan have been reported in several studies. Telmisartan prevented high fat diet-induced weight gain even in AT1 receptor deficient mice^31^, which suggests mechanism(s) independent of AT1 receptor blockade may play a role in anti-obesity effects of telmisartan. On the other hand, the weight loss effects of telmisartan were not observed in PPARδ knockout mice, which implies PPARδ activation by telmisartan is essential for its anti-obesity effects^30^. Our results also concur with those of He *et al*.^30^, regarding the involvement of PPARδ activation in telmisartan-induced browning but indirectly by PPARδ/γ-mediated M2 polarization.

Cold exposure is known to trigger adipose tissue browning and increase M2 macrophage contents in white adipose tissues^20^. Correspondingly, telmisartan administration to mice housed at 23 °C induced M2 polarization and subsequently adipose tissue browning, as evaluated by the histological analysis, OCR and the expression of thermogenic genes including UCP-1. On the other hand, treatment of thermoneutral mice with telmisartan also promoted adipose tissue browning, supporting pharmacological modulation of adipose tissue browning to treat obesity and its related metabolic disorders. In addition, since macrophage depletion by clodronate treatment suppressed the *in vivo* browning effects of telmisartan, it would appear M2 polarization at least partly contributes to the browning effects of telmisartan. Of note, administration of IL-4 complex also increased UCP-1 expression and OCR in parallel with M2 polarization in our experimental conditions (at 23 °C), supporting a critical role of M2 polarization in adipose tissue browning. Summarizing, the results obtained suggest telmisartan has therapeutic potential for the treatment of obesity and related metabolic disorders, and that its activity is associated with M2 polarization-induced adipose tissue browning. However, further study is required to elucidate in detail the mechanisms involved.

## Methods

### Materials

Dulbecco’s modified Eagle’s minimum essential medium (DMEM), fetal bovine serum (FBS), fetal calf serum (FCS), penicillin, and streptomycin were obtained from Gibco (Grand Island, NY). Monoclonal antibodies against ARG-1, TMEM26, PPARγ and *β*-actin were from Santa Cruz Biotechnology (Delaware, CA). Antibodies against UCP-1 and PPARδ were from Bioworld Technology (Minneapolis, MN). Peroxidase-conjugated secondary antibodies were from the Jackson Laboratory (Sacramento, CA), and enzyme immunoassay (EIA) kits for catecholamines were from R&D Systems (Minneapolis, MN). Random oligonucleotide primers and M-MLV reverse transcriptase were from Promega (Fitchburg, WI). TOPscriptTM RT Dry MIX was from Enzynomics (Daejeon, Korea). Thunderbird SYBR qPCR Mix was from Toyobo (Osaka, Japan). ARG-1, MRC-1, IL-10, UCP-1, Cited1, COX7a1, TMEM26 and GAPDH oligonucleotide primers were from Bioneer (Daejeon, Korea). Phenylmethylsulfonyl fluoride (PMSF), GW9662, GSK0660, telmisartan, triiodothyronine (T3) and all other chemicals were from Sigma Chemical (St. Louis, MO).

### Cell culture and differentiation

RAW 264.7 murine macrophage and 3T3/L1 pre-adipocytes cell lines were obtained from the Korean Cell Line Bank (Seoul, Korea), and grown at 37 °C in DMEM medium supplemented with 10% FCS (3T3/L1 pre-adipocytes) and FBS (RAW 264.7), penicillin (100 U/ml) and streptomycin sulfate (100 mg/ml) in a humidified 5% CO_2_ atmosphere. RAW 264.7 cells were treated with various concentrations (1, 10, 50 or 100 nM) of telmisartan for 24 h. To induce adipocyte differentiation, 3T3/L1 pre-adipocyte cells were cultured until confluent, and then exposed to differentiation medium (DMEM containing 0.25 mM 3-isobutyl-1-methylxanthine (IBMX), 0.25 μM dexamethasone, and 1 μg/ml insulin) for 2 days. Differentiated cells were cultured for another 2 days in DMEM containing 1 μg/ml insulin and 10% FBS, and then maintained in DMEM medium containing 10% FBS with media changes every other day for 6 days. To induce adipocyte browning, differentiated white adipocytes were supplemented with 50 nM tri-iodothyronine (T3) or Tel-CM (400 μl) from days 10 to 16, and again media were changed every other day.

### Primary bone marrow-derived macrophage (BMDM) culture and differentiation

BMDMs were isolated from 6 weeks old male C57BL/6J mice. Briefly, femurs of the mice were isolated after sacrifice by cervical dislocation, and marrow was obtained by irrigation with culture medium and dispersed by passing dispersions through a 25-gauge needle. After suspension and washing, the cells were cultured in 60 mm Petri dishes in DMEM containing 10% FBS as a source of macrophage colony-stimulating factor (M-CSF) at 37 °C in a humidified 5% CO_2_ atmosphere for 2 days. Cells were then maintained in DMEM containing 10% FBS for 8 days with medium changes every 2 days.

### Stromal vascular (SV) culture and adipocyte differentiation

To isolate murine adipose-derived cells, subcutaneous adipose tissues from male C57BL/6J mice (6 weeks of age) were finely minced, and digested using 2 mg/ml collagenase type XI (Sigma Chemical Co.) for 1 h at 37 °C. After centrifugation at 1200 x g for 10 min, SV fractions were resuspended in ammonium chloride (160 mM), incubated at room temperature for 10 min to lyse contaminating red blood cells, and then centrifuged again at 1200 × g for 10 min. SV cells were then cultured overnight in T25 flasks containing culture medium (DMEM/F12 containing 20% FBS, 1% penicillin/streptomycin) for 48 h at 37 °C in 5% CO_2_. After extensive washing with PBS to remove residual nonadherent cells, confluent SV cells were exposed to an adipogenic cocktail containing 1 μM dexamethasone, 10 μg/ml insulin, 0.5 mM isobutylmethylxanthine, and 200 μM indomethacin in culture medium for 2 days. Cells were then maintained in culture medium containing 10 μg/ml insulin for 14 days (day 16) with medium changes every 2 days. To induce browning, white adipocytes were cultured in DMEM supplemented with 50 nM T3 or Tel-CM from day 16 to 24 after with medium changes every 2 days.

### Determination of CA

RAW264.7 cells were treated with or without telmisartan for 24 h, and then levels of catecholamine in culture media were quantified using ELISA kits (Becton Dickinson, Franklin Lakes, NJ). Fresh culture media were used as blanks in all experiments.

### Immunocytochemistry

Differentiated 3T3/L1 adipocytes were treated with Tel-CM for 6 days, fixed with 100% methanol for 30 min, incubated with 0.1% Triton X-100 for 30 min and blocked with 5% normal goat serum for 1 h. Cells were then probed with mouse UCP-1 antibody (Abcam, diluted 1:500) overnight at 4 °C, incubated with goat anti-mouse 594 Alexa conjugated secondary antibody (Invitrogen, diluted 1:500) for 1 h at 24 °C, washed with PBS three times, mounted with mounting solution containing 4′,6-diamidino-2-phenylindole (DAPI), and then observed under a microscope.

### Mitotracker staining

Differentiated 3T3/L1 adipocytes were treated with Tel-CM for 6 days. The media were removed from the plate and add staining solution containing Mitotracker probe(100 nM, MitoTracker^®^ Red CMXRos, Cell signaling). Then the cells were incubated for 15–45 min under growth media and washed in fresh growth medium. The adipocytes were fixed with 3.7% formaldehyde in medium at 37 °C for 15 min and washed several times with PBS. The cells were mounted with mounting solution containing DAPI. Fluorescence images were captured by confocal laser scanning microscope LSM 700 (Carl Zeiss, Oberkochen, Germany). Mitotracker stainings of adipose tissues were done in a similar way to those of adipocytes.

### Western blot analysis

For western blot analysis, treated cells were washed with PBS once and then collected by centrifuge. The cells were then lysed with extraction lysis buffer PRO-PREP (Intron Biotechnology, Sungnam, Korea), incubated for 30 min on ice, and centrifuged at 14,000 rpm for 30 min at 4 °C. Protein concentrations were determined using Bio-Rad protein assay reagent according to the manufacturer’s instructions. Cellular proteins (30 μg) from treated or untreated cell extracts were separated by 10% SDS-polyacrylamide gel electrophoresis, and then electroblotted onto PVDF membranes. Immunoblots were incubated overnight with blocking solution (2% BSA) at 4 °C, with primary antibody (Santa Cruz Biotechnology) overnight, washed four times with Tween 20/Tris-buffered saline (T-TBS), and incubated with horseradish peroxidase-conjugated secondary antibody (1:1000; Santa Cruz Biotechnology) for 1 h at room temperature. Blots were again washed three times with T-TBS, and developed by enhanced chemiluminescence (Amersham Life Science). All protein levels were normalized versus β-actin.

### RNA preparation and real-time PCR

Total RNAs were extracted from cells using Easy Blue kits (Intron Biotechnology). RNAs were converted to cDNAs using TOPscriptTM RT Dry MIX (Enzynomics, Daejeon, Korea). Real-time PCR reactions were run in duplicate for each sample, and transcript levels of each gene were normalized versus GAPDH. The primer sequences used were as follows: ARG-1 sense-5′- AGA GAC CAC GGG GAC CTG GC -3′ and antisense-5′- TGG ACC TCT GCC ACC ACA CC-3′, MRC-1 sense-5′- CTC TGT TCA GCT ATT GGA CGC-3′ and antisense-5′- CGG AAT TTC TGG GAT TCA GCT TC-3′, IL-10 sense-5′- GCT GGA CAA CAT ACT GCT AAC C-3′ and antisense-5′- ATT TCC GAT AAG GCT TGG CAA-3′, UCP-1 sense-5′- ACT GCC ACA CCT CCA GTC AT-3′ and antisense-5′- CTT TGC CTC ACT CAG GAT TG-3′, Cited1 sense-5′- CGC TTC GTC CGT ACC TCA GC-3′ and antisense-5′- CAG CTG GGC CTG TTG GTC TC-3′, COX7a1 sense-5′- AAA GTG CTG CAC GTC CTT G-3′ and antisense-5′- TTC TCT GCC ACA CGG TTT TC-3′, TMEM26 sense-5′- TCC TGT TGC ATT CCC TGG TC-3′ and antisense-5′- GCC GGA GAA AGC CAT TTG T-3′, and GAPDH sense-5′- AGG TCG GTG TGA ACG GAT TTG-3′ and antisense-5′-GGG GTC GTT GAT GGC AAC A-3′, PPARγ sense-5′-CAT CCA AGA CAA CCT GCT GC-3′ and antisense-5′-TGT GAC GAT CTG CCT GAG GT-3′, and PPARδ sense-5′-GCT GCT GCA GAA GAT GGC A-3′ and antisense-5′-CAC TGC ATC ATG TGG GCA TG-3′.

### FACS analysis

FACS analysis was performed using a BD FACS Calibur (BD Biosciences), and data were processed and analyzed using Flowjo software (Treestar, Ashland, OR). Isolation of macrophages (F4/80) and M2 (CD206) was performed using a FACS Aria machine (BD Biosciences), using the following antibodies: FITC-conjugated anti-mouse F4/80 (Biolegend) and FITC-conjugated IgG2a rat isotype control, and APC-conjugated anti-mouse CD206 and APC-conjugated IgG2a rat isotype control (Biolegend).

### Animals

Male C57BL/6J (6 weeks old) were obtained from Orient Bio (Seoul, Korea), and acclimated for one week under constant conditions (temperature: 23 ± 2 °C, humidity: 40–60%, under a 12 h light/dark cycle), and allowed *ad libitum* access to a standard chow diet and tap water. For experiments, mice were randomized into three groups (n = 6 each group): Group 1 (the control group) animals were treated with vehicle (0.9% saline); Group 2 were treated with telmisartan (1 mg/kg); and Group 3 were treated with telmisartan (3 mg/kg) by oral injection once daily for 2 weeks. Body weights, food intakes and blood glucose levels were measured weekly at the same time every week (between 10:00 and 11:00 AM) for 2 weeks. Food intakes were determined by subtracting food amounts left in cages from the amounts supplied (n = 6 in each cage). Blood glucose concentrations were determined in tail vein blood using an Allmedicus Gluco Dr. Plus (Seoul, Korea) in the fed state. After 2 weeks administration of telmisartan, one set was subjected to OGTT after a 16 h fast (from 6:00 PM to 10:00 AM the next day), during which only water was supplied. Briefly, animals were administered d-glucose (2 g/kg) orally, and blood glucose levels were measured in tail blood at 15, 30, 60 and 90 min after glucose administration using an Allmedicus Gluco Dr. Plus (Seoul). The area under the curve (AUC) for glucose calculated using OriginPro 6.1 software (Origin, Northampton, MA). After OGTT, the mice were anesthetized by CO_2_ inhalation, and adipose tissues were rapidly isolated and weighed. Adipose tissues from one side were frozen in liquid nitrogen and stored at −80 °C until required for further analyses. The middle portions of other side were fixed in 4% paraformaldehyde for sectioning and staining. After two weeks treatment of telmisartan, another set was subjected to cold exposure for 48 h, during which rectal temperatures were measured, and *ex vivo* OCR and Mitotracker staining were determined after sacrifice. To compare the effects of telmisartan with a known M2 inducer, IL-4 (2 μg) complexed with anti-IL-4 antibody (10 μg, 1:5) were separately injected intraperitoneally (100 μl/mice, n = 8) every other day for 2 weeks either at 23 ± 2 °C or 30 ± 2 °C. One set was subjected to OGTT followed by adipose tissue analysis, and another set was subjected to cold exposure.

For experiments under thermoneutral condition, mice were acclimated for one week under constant conditions (temperature: 30 ± 2 °C, humidity: 40–60%, under a 12 h light/dark cycle), and allowed *ad libitum* access to a standard chow diet and tap water. Then, mice were randomized into three groups (n = 6 each group): Group 1 (the control group) animals were treated with vehicle (0.9% saline); Group 2 were treated with telmisartan (1 mg/kg); and Group 3 were treated with telmisartan (3 mg/kg) by oral injection once daily for 2 weeks under the temperature of 30 ± 2 °C. Following procedures were the same as described above. All animal procedures were performed in accordance with the Guide for the Care and Use of Laboratory Animals published by the US National Institute of Health (NIH Publication No. 85-23, revised 2011) and approved by the Animal Care and Use Committee of Gachon University.

### Macrophage depletion with clodronate liposome

Control liposomes (F70101-N) and clodronate liposomes (F70101C-N, 200 μl, Sunnyvale, CA) were given intraperitoneally to C57BL/6J mice (7 weeks old, male, n = 8 in each group) once every other day for 2 weeks along with daily administration of telmisartan (3 mg/kg). The extent of browning by telmisartan was determined by real time qPCR of browning markers in control and clodronate liposome-treated adipose tissues. Macrophage depletion by clodronate liposomes was confirmed by the expressions of F4/80 and CD11b (representative macrophage markers).

### O_2_ consumption rate assay

*In vitro* O_2_ consumption was measured using a Mito-ID^®^ O_2_ extracellular sensor kit (Enzo, Farmingdale, NY). White adipocytes were treated with Tel-CM to induce browning. After 6 days treatment, cells were placed in fresh medium for 30 min, treated with dissolved oxygen (DO) solution for 30 min, and O_2_ concentrations were measured. To measure oxygen concentrations in adipose tissues, tissue slices (3 × 3 mm) from vehicle or telmisartan-treated mice were cultured in DMEM medium for 24 h and treated with DO solution for 30 min before oxygen concentrations were measured.

### Histopathology

Adipose tissues were fixed in 4% paraformaldehyde overnight and embedded in paraffin. Immunohistochemical detection of UCP-1 or ARG-1 was carried out using the avidin-biotin-DAB complex method on paraffin sections. Briefly, after an overnight incubation at 4 °C with primary monoclonal antibodies against UCP-1 or ARG-1 (Santa Cruz Biotechnology, diluted 1:50), a biotin-conjugated goat anti-rabbit secondary antibody (Vector Laboratories, Burlingame, CA) diluted 1:250), and subsequently streptavidin-conjugated with horseradish peroxidase (Vector Laboratories, diluted 1:250) was applied. DAB peroxidase substrate (Vector Laboratories) was used for visualization, and the specimens were counterstained with hematoxylin (Sigma Chemical).

### Statistical analysis

The significances of differences versus respective controls were determined using the Student’s *t*-test for paired experiments or two-way ANOVA. Results are presented as the means ± SDs of three separate experiments, and *p* values of < 0.05 were considered statistically significant.

## Supplementary information

Supplementary information file

## Data Availability

The datasets and reagents generated during the current study are available from the corresponding author on reasonable request.

## References

[1] Skeggs LT, Dorer FE, Kahn JR, Lentz KE, Levine M (1976). The biochemistry of renin angiotensin system and its role in hypertension. Am. J. Med..

[2] Kjeldsen SE, Julius S (2004). Hypertension mega-trials with cardiovascular end points: effect of angiotensin-converting enzyme inhibitors and angiotensin blockers. Am. Heart J..

[3] Sharma AM, Janke J, Gorzelniak K, Engeli S, Luft FC (2002). Angiotensin blockade prevents type 2 diabetes by formation of fat cells. Hypertension.

[4] Schling P, Loffler G (2001). Effects of angiotensin II on adipose conversion and expression of genes of the rennin-angiotensin system in human preadipocytes. Horm. Metab. Res..

[5] Araki K (2006). Telmisartan prevents obesity and increases the expression of uncoupling protein 1 in diet-induced obese mice. Hypertension.

[6] Dinh DT, Frauman AG, Johnston CI, Fabiani ME (2001). Angiotensin receptors: distribution, signaling and function. Clin. Sci. (Lond.).

[7] Benson SC (2004). Identification of telmisartan as a unique angiotensin II receptor antagonist with selective PPARγ modulating activity. Hypertension.

[8] Staels B, Fruchart JC (2005). Therapeutic roles of peroxisome proliferator-activated receptor agonists. Diabetes.

[9] Berger JP (2003). Distinct properties and advantages of a novel peroxisome proliferator-activated protein γ selective modulator. Mol. Endocrinol..

[10] O’Shea JJ, Paul WE (2010). Mechanisms underlying lineage commitment and plasticity of helper CD4+ T cells. Science.

[11] Thomsen LH, Rosendahl A (2015). Polarization of macrophages in metabolic diseases. J. Clin. Cell Immunol..

[12] Kanda H (2006). MCP-1 contributes to macrophage infiltration into adipose tissue, insulin resistance, and hepatic steatosis in obesity. J. Clin. Invest..

[13] Weisberg SP (2003). Obesity is associated with macrophage accumulation in adipose tissue. J. Clin. Invest..

[14] Castoldi A, Naffah de Souza C, Camara NO, Moraes-Vieira PM (2016). The macrophage switch in obesity development. Front. Immunol..

[15] Gordon S (2003). Alternative activation of macrophages. Nat. Rev. Immunol..

[16] Zhou D (2014). Macrophage polarization and function with emphasis on evolving roles of coordinated regulation of cellular signaling pathways. Cell. Signal..

[17] Odegaard JI (2007). A. Macrophage specific PPARγ controls alternative activation and improves insulin resistance. Nature.

[18] Bouhlel MA (2007). PPARγ activation primes human monocytes into alternative M2 macrophages with anti-inflammatory properties. Cell Metab..

[19] Langin D (2010). Recruitment of brown fat and conversion of white into brown adipocytes: strategies to fight the metabolic complications of obesity. Biochem. Biophys. Acta.

[20] Nguyen KD (2011). Alternatively activated macrophages produce catecholamines to sustain adaptive thermogenesis. Nature.

[21] Sugimoto K (2006). Telmisartan but not valsartan increases caloric expenditure and protects against weight gain and hepatic steatosis. Hypertension.

[22] Fujisaka S (2011). Telmisartan improves insulin resistance and modulates adipose tissue macrophage polarization in high fat-fed mice. Endocrinology.

[23] Liu PS, Lin YW, Burton FH, Wei LN (2015). Injecting engineered anti-inflammatory macrophages therapeutically induces white adipose tissue browning and improves diet-induced insulin resistance. Adipocyte.

[24] Qiu Y (2014). Eosinophils and type 2 cytokine signaling in macrophages orchestrate development of functional beige fat. Cell.

[25] Engeli S (2003). The adipose tissue renin angiotensin-aldosterone system: role in metabolic syndrome?. Int. J. of Biochem. Cell Biol..

[26] Goossens GH, Blaak EE, van Baak MA (2003). Possible involvement of the adipose tissue renin-angiotensin system in pathophysiology of obesity and obesity-related disorders. Obes. Rev..

[27] Tsukuda Kana, Mogi Masaki, Iwanami Jun, Kanno Harumi, Nakaoka Hirotomo, Wang Xiao-Li, Bai Hui-Yu, Shan Bao-Shuai, Kukida Masayoshi, Higaki Akinori, Yamauchi Toshifumi, Min Li-Juan, Horiuchi Masatsugu (2016). Enhancement of Adipocyte Browning by Angiotensin II Type 1 Receptor Blockade. PLOS ONE.

[28] Graus-Nunes F, Rachid TL, de Oliveira Santos F, Barbosa-da-Silva S, Souza-Mello V (2016). AT1 receptor antagonist induces thermogenic beige adipocytes in the inguinal white adipose tissue of obese mice. Endocrine.

[29] Furuhashi M (2004). Blockade of the renin–angiotensin system decreases adipocyte size with improvement in insulin sensitivity. J. Hypertens..

[30] He H (2010). Telmisartan prevents weight gain and obesity through activation of peroxisome proliferator-activated receptor δ-dependent pathways. Hypertension.

[31] Rong X (2010). Angiotensin II type 1 receptor-independent beneficial effects of telmisartan on dietary-induced obesity, insulin resistance and fatty liver in mice. Diabetologia.

[32] Kolli V (2014). Partial agonist, telmisartan, maintains PPARr serine 112 phosphorylation, and dose not affect osteoblast differentiation and bone mass. Plos One.

[33] Peschechera A, Eckel J (2013). Browning of adipose tissue-regulation and therapeutic perspectives. Arch. Physiol. Biochem..

[34] Bartelt A, Heeren J (2014). Adipose tissue browning and metabolism. Nature.

[35] Bonet ML, Oliver P, Palou A (2013). Pharmacological and nutritional agents promoting browning of white adipose tissue. Biochim. Biophys. Acta.

[36] Hui X (2015). Adiponectin enhances cold-induced browning of subcutaneous adipose tissue via promoting M2 macrophage proliferation. Cell Metab..

[37] Brestoff JR (2015). Group 2 innate lymphoid cells promote beiging of white adipose tissue and limit obesity. Nature.

[38] Fischer K (2017). Alternatively activated macrophages do not synthesize catecholamines or contribute to adipose tissue adaptive thermogenesis. Nat. Med..

[39] Mukundan L (2009). PPAR delta senses and orchestrates clearance of apoptotic cells to promote tolerance. Nat. Med..

[40] Filippo, C. D. *et al*. Involvement of proteasome and macrophages M2 in the protection afforded by telmisartan against the acute myocardial infarction in Zucker diabetic fatty rats with metabolic syndrome. *Mediators Inflamm*. 1–9 (2014). 10.1155/2014/972761 PMC4119687 25110402

[41] Xu Y (2015). Telmisartan prevention of LPS-induced microglia activation involved M2 microglia polarization via CaMKKβ–dependent AMPK activation. Brain, Behav. Immun..

[42] Shiota A (2012). Telmisartan ameliorates insulin sensitivity by activating the AMPK/SIRT1 pathway in skeletal muscle of obese db/db mice. Cardiovasc. Diabetol..

[43] Shiota A (2012). Activation of AMPK-Sirt1 pathway bt telmisartan in white adipose tissue: A possible link to anti-metabolic effects. Eur. J. Pharmacol..

