# Author Correction: Telmisartan induces browning of fully differentiated white adipocytes via M2 macrophage polarization

**DOI:** 10.1038/s41598-020-58948-x

**Published:** 2020-02-04

**Authors:** Eun Jeong Jeon, Dong Young Kim, Na Hyun Lee, Hye-Eun Choi, Hyae Gyeong Cheon

**Affiliations:** 1 Department of Pharmacology, Gachon University School of Medicine, Incheon, 21999 Republic of Korea; 2 Department of Health Sciences and Technology, GAIHST, Gachon University, Incheon, 21999 Republic of Korea

Correction to: *Scientific Reports* 10.1038/s41598-018-38399-1, published online 04 February 2019

This Article contains an error in Figure 8f, where the incorrect image was used for the telmisartan treatment (1 mg/kg) in brown fat tissue. The correct Figure 8 appears below.

**Figure 1:**
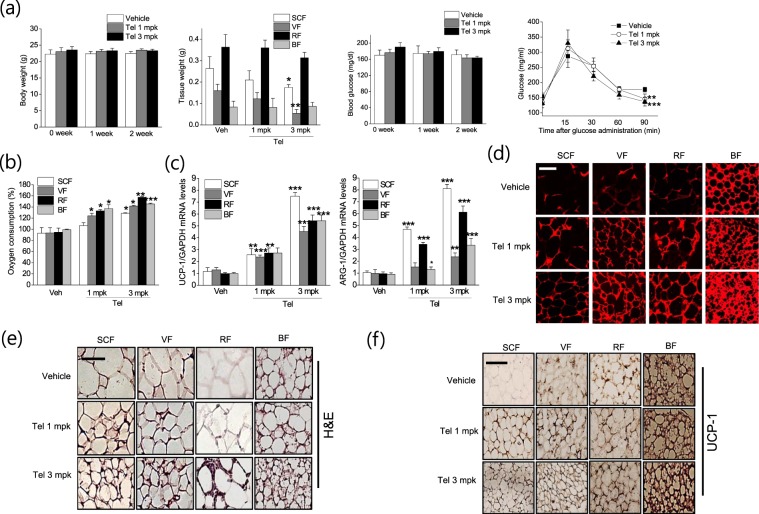
.

