# Supplementary material for: Telmisartan induces browning of fully differentiated white adipocytes via M2 macrophage polarization
Source: Sci Rep. 2019 Feb 4;9:1236. doi: 10.1038/s41598-018-38399-1 (PMC6362091; doi:10.1038/s41598-018-38399-1)
Supplement: Supplementary file 1 — Supplementary information file [file 41598_2018_38399_MOESM1_ESM.pdf]

Telmisartan induces browning of fully differentiated white adipocytes via M2 macrophage polarization

Eun Jeong Jeon, Dong Young Kim, Na Hyun Lee, Hye-Eun Choi, Hyae Gyeong Cheon*

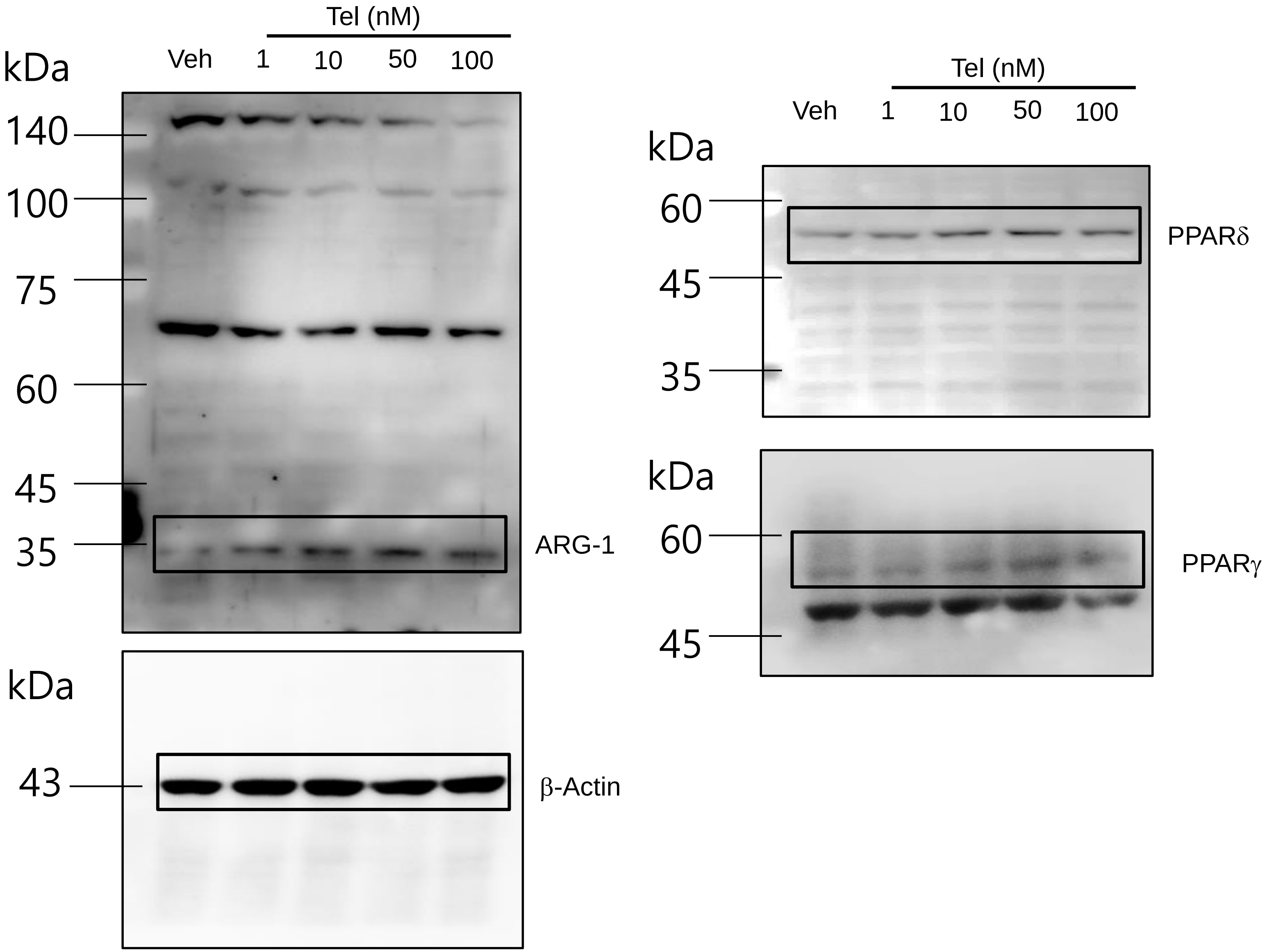

Supplementary Figure 1. Uncropped scans of western blot displayed in Fig. 1b

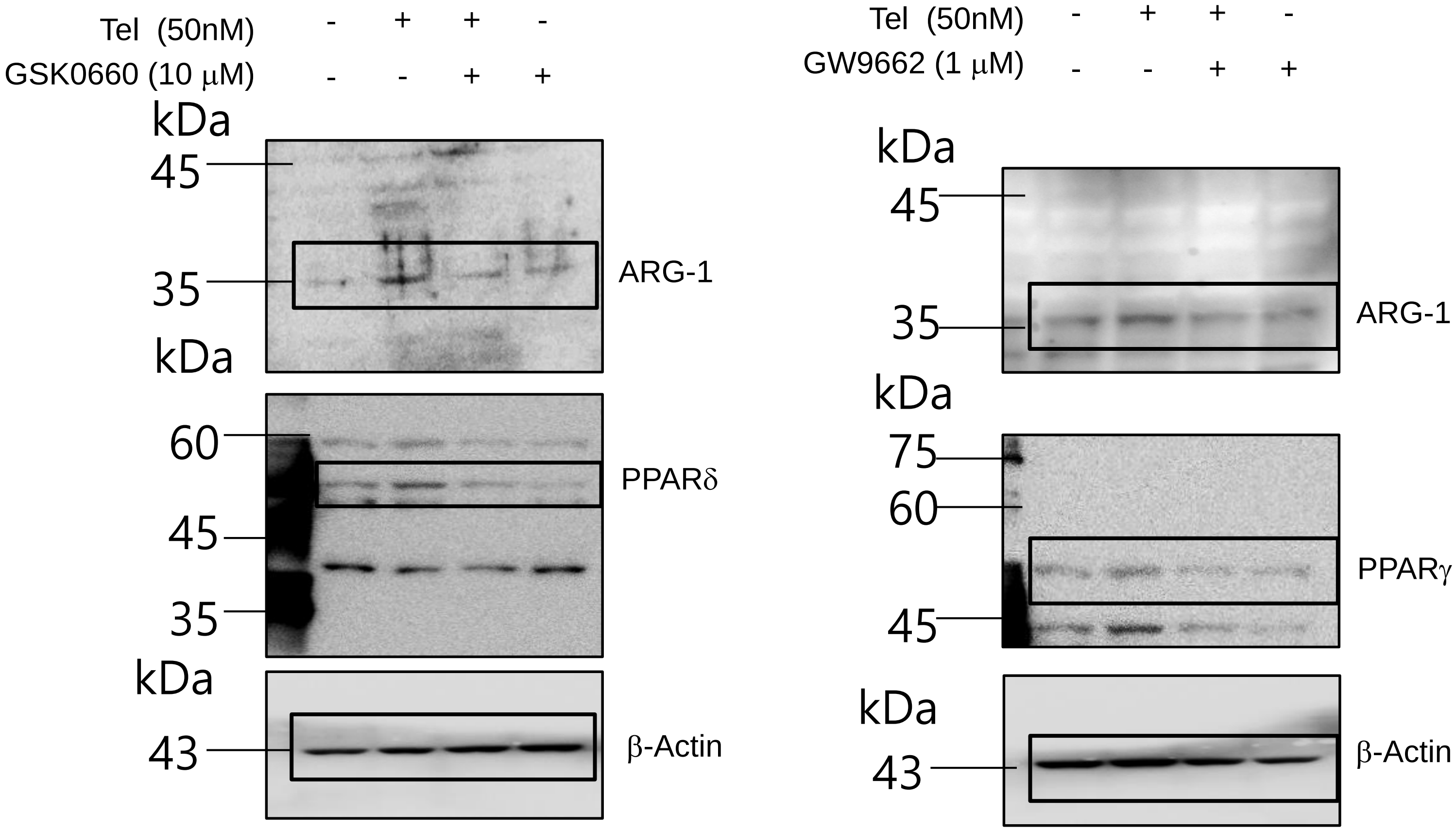

Supplementary Figure 2. Uncropped scans of western blot displayed in Fig. 3c

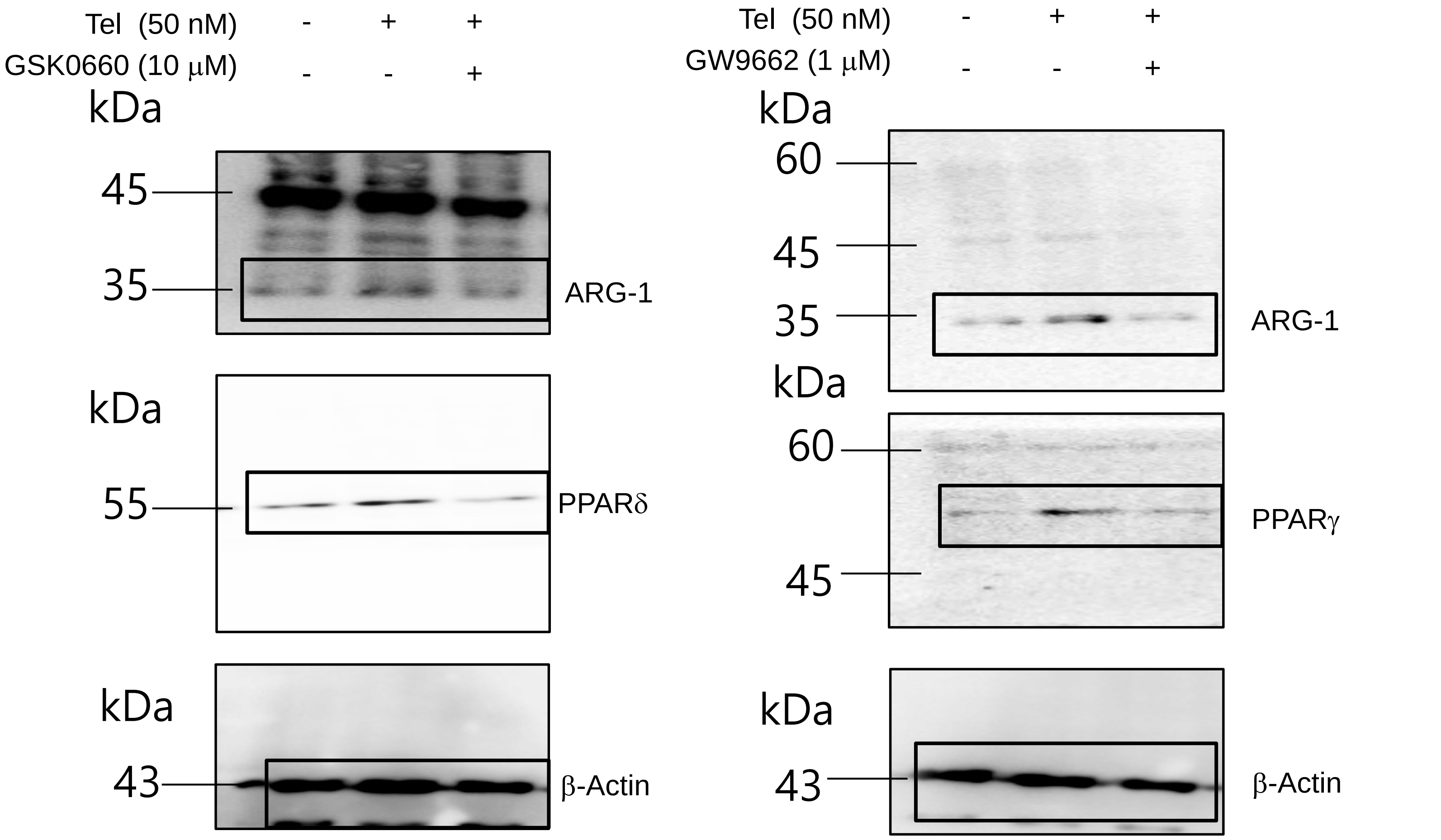

Supplementary Figure 3. Uncropped scans of western blot displayed in Fig. 3d

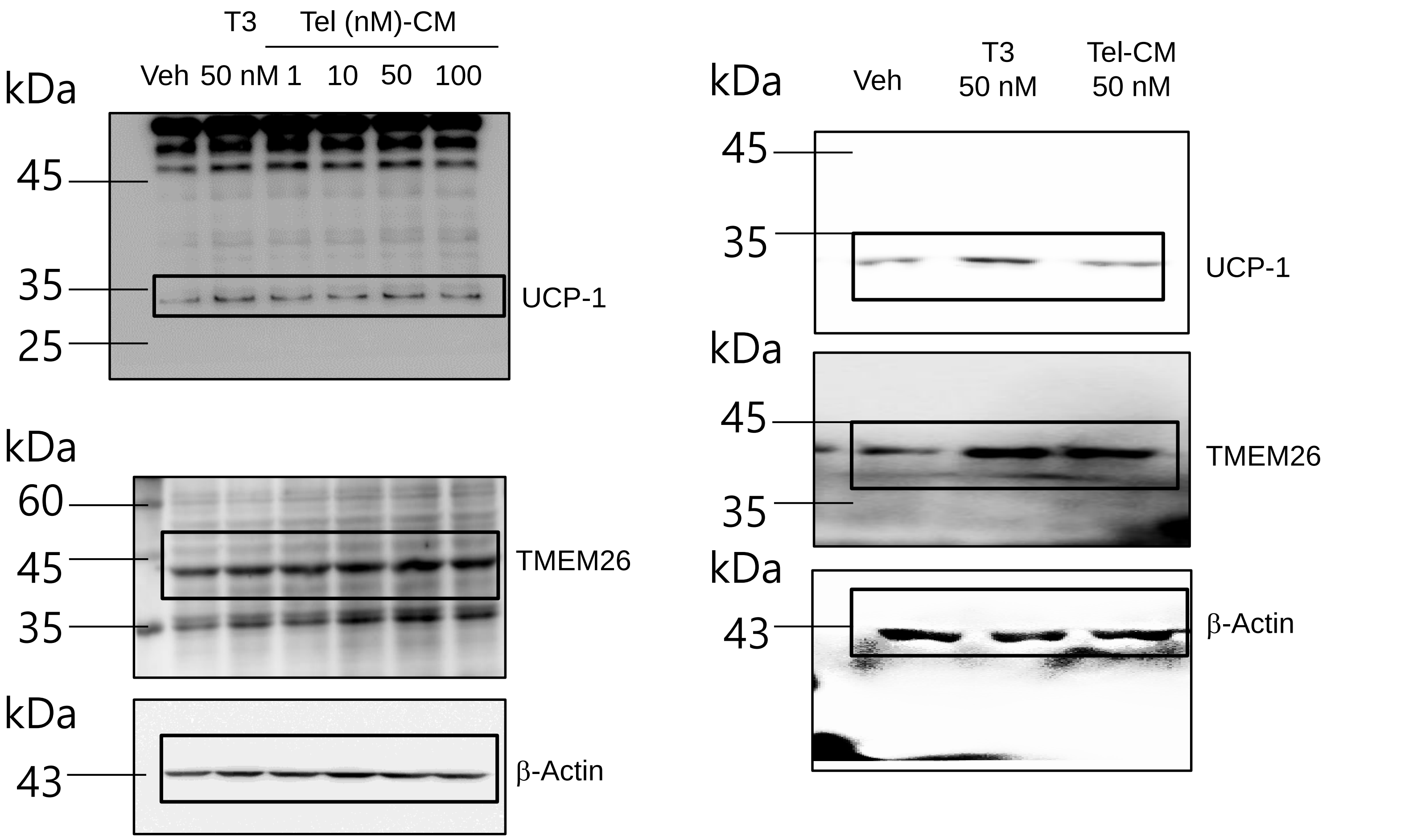

Supplementary Figure 4. Uncropped scans of western blot displayed in Fig. 4b, and e
